# Substrate stiffness controls proinflammatory responses in human gingival fibroblasts

**DOI:** 10.1038/s41598-023-28541-z

**Published:** 2023-01-24

**Authors:** Watcharaphol Tiskratok, Masahiro Yamada, Jun Watanabe, Nadia Kartikasari, Tsuyoshi Kimura, Hiroshi Egusa

**Affiliations:** 1 Division of Molecular and Regenerative Prosthodontics, Tohoku University Graduate School of Dentistry, Sendai, Miyagi Japan; 2 Institute of Dentistry, Suranaree University of Technology, Mueang Nakhon Ratchasima District, Nakhon Ratchasima Thailand; 3 Institute of Biomaterials and Bioengineering, Tokyo Medical and Dental University, Chiyoda-Ku, Tokyo, Japan; 4 Center for Advanced Stem Cell and Regenerative Research, Tohoku University Graduate School of Dentistry, Sendai, Miyagi Japan

**Keywords:** Infection, Myosin, Periodontics

## Abstract

Soft gingiva is often compromised in gingival health; however, the underlying biological mechanisms remain unknown. Extracellular matrix (ECM) stiffness is involved in the progression of various fibroblast-related inflammatory disorders via cellular mechanotransduction. Gingival stiffness might regulate cellular mechanotransduction-mediated proinflammatory responses in gingival fibroblasts. This in vitro study aims to investigate the effects of substrate stiffness on proinflammatory responses in human gingival fibroblasts (hGFs). The hGFs isolated from two healthy donors cultured on type I collagen-coated polydimethylsiloxane substrates with different stiffnesses, representing soft (5 kPa) or hard (25 kPa) gingiva. Expression levels of proinflammatory mediators, prostaglandin E2 or interleukin-1β, in hGFs were significantly higher with the soft substrate than with the hard substrate, even without and with lipopolysaccharide (LPS) to induce inflammation. Expression levels of gingival ECM and collagen cross-linking agents in hGFs were downregulated more with the soft substrate than with the hard substrate through 14 days of culture. The soft substrate suppressed the expression of mechanotransduction-related transcriptional factors and activated the expression of inflammation-related factors, whereas the hard substrate demonstrated the opposite effects. Soft substrate induced proinflammatory responses and inhibition of ECM synthesis in hGFs by inactivating cellular mechanotransduction. This supports the importance of ECM stiffness in gingival health.

## Introduction

The gingival phenotype is a clinical parameter used to assess the structural rigidity of the attached gingiva^1^ and is closely related to the prognosis of restorative, prosthodontic, and implant therapies^2^. Thick gingiva is considered to resist gingival recession and periodontitis progression, while thin and soft gingiva tends to show high susceptibility to the disruption of periodontal health^3^. However, the underlying biological mechanisms responsible for the gingival phenotype have not yet been elucidated.

Fibroblasts are the predominant resident cells in gingival connective tissues and play important roles in their maintenance, repair, and regeneration^4^. Fibroblasts are involved in the degradation of the gingival fibrous structure via the secretion of matrix metalloproteinases (MMPs) and pro-inflammatory mediators such as interleukin-1β or -6^5^. Furthermore, human gingival fibroblasts (hGFs) may modulate the proinflammatory responses of macrophages to lipopolysaccharide (LPS) in the gingiva^6^.

Cellular mechanotransduction is a complex cellular process that regulates the cell fate and function^7^ by converting a mechanical stimulus of the local environment into biochemical signals with mechano-sensors, such as a focal adhesion plaque^8^ or the calcium ion channel, Piezo1^9^. The stiffness of the local environment, such as the extracellular matrix (ECM), regulates fibroblast attachment^10^, proliferation, and ECM formation^11^, leading to structural changes in the ECM that induce further feedback regulation of fibroblast function^7,12^. This feedback loop regulation between fibroblasts and the ECM is involved in various pathological conditions, including wound healing^13^, tissue fibrosis^14^, and tumor progression^15^. Matrix stiffness may be associated with the activation of Toll-like receptors (TLRs) in certain types of fibroblasts^16^ that govern collagen synthesis for early wound healing or skin fibrosis^17^. Furthermore, the stiffness of the microenvironment has the potential to tune cellular proinflammatory responses via yes-associated protein (YAP)-mediated mechanotransduction^18^. A similar phenomenon is observed in oral tissue-derived cells. It has been proposed that oral mucosal fibroblasts may sense mechanical stress to influence alveolar bone resorption in cooperation with immune cells by regulating production of cytokines, including prostaglandin E2 (PGE_2_)^19^. Indeed, cyclic pressure induces proinflammatory responses in hGFs to stimulate osteoclast differentiation^20^. The osteogenic differentiation of human periodontal ligament cells declines with a reduction in substrate stiffness^21^. Substrate stiffness modulates proinflammatory responses in human gingiva-derived mesenchymal stem cells^22^.

Based on this background, we hypothesized that soft gingival ECM facilitated proinflammatory responses and inhibition of ECM synthesis in hGFs via cellular mechanotransduction; therefore, ECM stiffness mediated the feedback loop regulation in hGFs. The objective of this study was to determine whether substrate stiffness regulated the proinflammatory responses and ECM synthesis in hGFs via cellular mechanotransduction.

## Results

### Substrate stiffness regulates proinflammatory responses in hGFs

**Figure 1:**
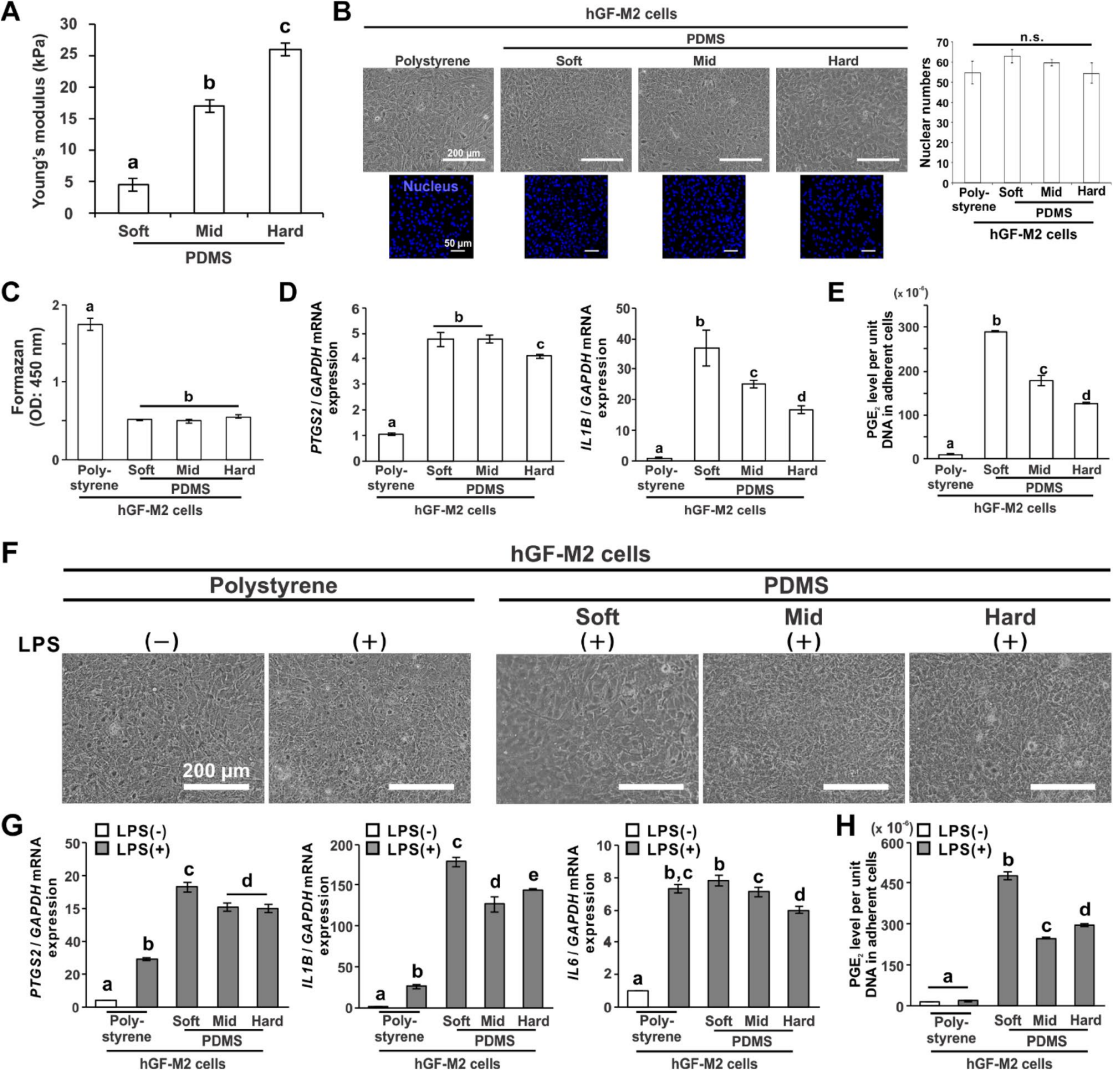
Effects of substrate stiffness on the proinflammatory responses of hGFs. (**A**) Results of Young’s modulus of PDMS substrates of soft, mid, and hard stiffness, respectively. (**B**,**F**) Representative phase microscopic images, (**B**) fluorescence confocal laser microscopic images of nucleus (blue) and the quantification of nuclear numbers based on images, (**C**) water-soluble tetrazolium (WST)-1-based colorimetry for cell attachment numbers, and (**D**,**G**) reverse transcription-polymerase chain reaction (RT-PCR)-based gene expression analysis for *PTGS2*, *IL1B* and *IL6* relative to *GAPDH* in the hGF-M2 cells cultured on the 0.1wt% collagen-coated polystyrene culture plate and soft, mid, and hard PDMS for 12 h with or without co-incubation with 1000 ng/mL of LPS. (**E**,**H**) PGE_2_ levels per unit DNA in adherent hGF-M2 cells under the corresponding culture condition as above by enzyme-linked immunosorbent assay (ELISA) analysis of culture supernatants. Data are represented as the means ± standard deviation (SD; *N* = 4 in B, *N* = 3 in C-E, G and H). Different letters indicate the statistically significant difference between them (*P* < 0.05; Tukey’s honestly significant difference [HSD] test). *hGFs* human gingival fibroblasts, *LPS* lipopolysaccharide, *PDMS* polydimethylsiloxane, *PGE*_*2*_ prostaglandin E2, *PTGS2* prostaglandin G/H synthase 2, *IL1B* Interleukin-1β, *IL6* Interleukin-6, *GAPDH* glyceraldehyde-3-phosphate dehydrogenase, *n.s.* non-significant difference.

Two populations of hGFs isolated from two different healthy donors were labeled hGF-M2 and hGF-F1 and cultured on polydimethylsiloxane (PDMS) substrates with various stiffness. Young’s modulus of the soft, mid, and hard PDMS substrates demonstrated 4.4, 17.0, or 26.2 kPa, respectively (Fig. 1A) (*P* < 0.05, Tukey’s honestly significant difference [HSD]). We confirmed that coating with 0.1wt% bovine dermis-derived native type I collagen solution enabled hGF cell attachment on the PDMS substrate (Fig. S1A). hGF-M2, 12 h after seeding, enabled adherence to all types of substrates (Fig. 1B). The number of nuclear and adherent cells on the PDMS substrates did not differ regardless of the stiffness (Fig. 1B,C). The expression levels of proinflammatory gene markers, prostaglandin G/H synthase 2 (*PTGS2)* and interleukin-1 $\beta$ (*IL1B)*, in hGF-M2 cells on the PDMS substrate were higher than those on polystyrene but decreased with increasing PDMS stiffness (Fig. 1D) (*P* < 0.05, Tukey’s HSD test). Soft PDMS had the highest gene expression among the substrates (*P* < 0.05, Tukey’s HSD test). The PGE_2_ levels were markedly higher on the PDMS substrates than on the polystyrene substrates (Fig. 1E) (*P* < 0.05, Tukey’s HSD test), but decreased with increasing PDMS stiffness, with the highest value on the soft PDMS (*P* < 0.05, Tukey’s HSD test).

Similar to the culture on polystyrene without LPS, the hGF-M2 cell cultures co-incubated with LPS for 12 h became confluent regardless of the type of substrate (Fig. 1F). However, the *PTGS2*, *IL1B*, and Interleukin-6 (*IL6)* gene expression levels were markedly upregulated in cultures co-incubated with LPS, particularly on PDMS substrates (Fig. 1G and Fig. S2C). Soft PDMS had the highest gene expression among the substrates (*P* < 0.05, Tukey’s HSD test). Likewise, the PGE_2_ levels were markedly higher on the PDMS substrates than on polystyrene substrates with or without LPS co-incubation, and the soft PDMS increased the value by over 50% of that on the other PDMS substrates (Fig. 1H) (*P* < 0.05, Tukey’s HSD test). Even in a different cell population, the hGF-F1 cells, the soft PDMS upregulated *IL1B* expression and further augmented the elevation of proinflammatory gene and PGE_2_ levels induced by LPS (Fig. S2). The levels of LPS-induced proinflammatory responses in the hGF-F1 cells gradually decreased to a level comparable to that of polystyrene with increasing PDMS stiffness (*P* < 0.05, Tukey’s HSD test).

**Figure 2:**
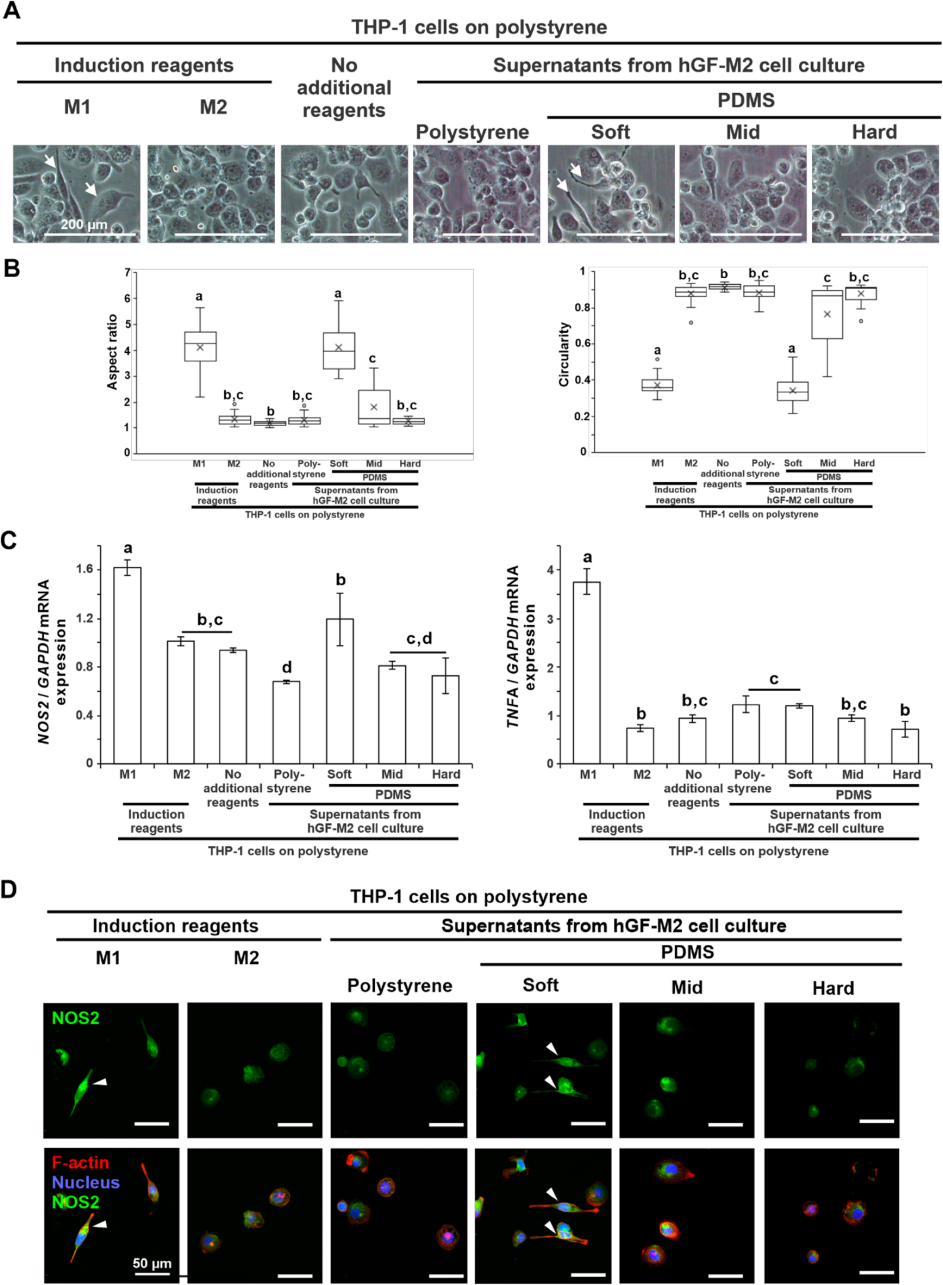
Effects of substrate stiffness on the immunostimulatory activity of hGFs. (**A**) Representative phase microscopic images, (**B**) the corresponding cytomorphometric parameters, aspect ratio and circularity, (**C**) reverse transcription-polymerase chain reaction (RT-PCR)-based gene expression analysis of *NOS2* and *TNFA* relative to *GAPDH* and (**D**) immunofluorescence confocal laser microscopic images of NOS2 (green), *F*-actin (red), and nucleus (blue) in THP-1 cell cultures on a polystyrene culture plate for 24 h via 12 h co-incubation with or without 50 ng/mL LPS and 20 ng/mL interferon $\gamma$ for M1-induction, 20 ng/mL interleukin-4 and 20 ng/mL interleukin-13 for M2-induction or the culture supernatant from hGF-M2 cells on the 0.1wt% collagen-coated polystyrene culture plate and soft, mid and hard PDMS. Note phase microscopic images (**A**) showing elongated spindle cells (arrows) in THP-1 cells with M1-induction or the supernatant from hGF-M2 cells on the soft PDMS. Data are presented as box plots (*N* = 20 in B) or the means ± standard deviation (SD; *N* = 3 in C). Different letters indicated the statistically significant difference between them (*P* < 0.05; Tukey’s honestly significant difference [HSD] test). Note: (D) elongated THP-1 cells with strong NOS2 signals (triangles) were observed in the THP-1 cell culture with M1-induction or the supernatant from hGF-M2 cells on the soft PDMS. *THP-1* human monocytic leukemia cell line, *PDMS* polydimethylsiloxane, *M1, M1* macrophage cells, *M2, M2* macrophage cells, *LPS* lipopolysaccharide, *NOS2* Nitric oxide synthase 2, *TNFA* tumor necrosis factor-α, *GAPDH* glyceraldehyde-3-phosphate dehydrogenase.

Elongated spindle cells in human monocytic leukemia cell line, THP-1, culture emerged only after co-culturing with M1-inducing reagents or with supernatants from hGF-M2 cells on soft PDMS (Fig. 2A, arrows). The aspect ratio or circularity were significantly higher or lower, respectively, in THP-1 cells after co-culturing with M1-inducing reagents and with supernatants from hGF-M2 cells, respectively, on soft PDMS than those with the other cultures (Fig. 2B). The hGF-M2 culture supernatants on the soft PDMS upregulated M1 macrophage markers, nitric oxide synthase 2 (*NOS2*), and tumor necrosis factor-alpha (*TNFA*), to a greater extent than the supernatants from the other PDMS substrates (Fig. 2C) (*P* < 0.05, Tukey’s HSD test), although not as high as those of the M1 macrophage-induced cells. Furthermore, fluorescent signals for NOS2 were highly detected in the elongated THP-1 cells co-cultured with M1-inducing reagents or with supernatants from hGF-M2 cells on soft PDMS (Fig. 2D, triangles).

### Substrate stiffness regulates ECM production of hGFs

**Figure 3:**
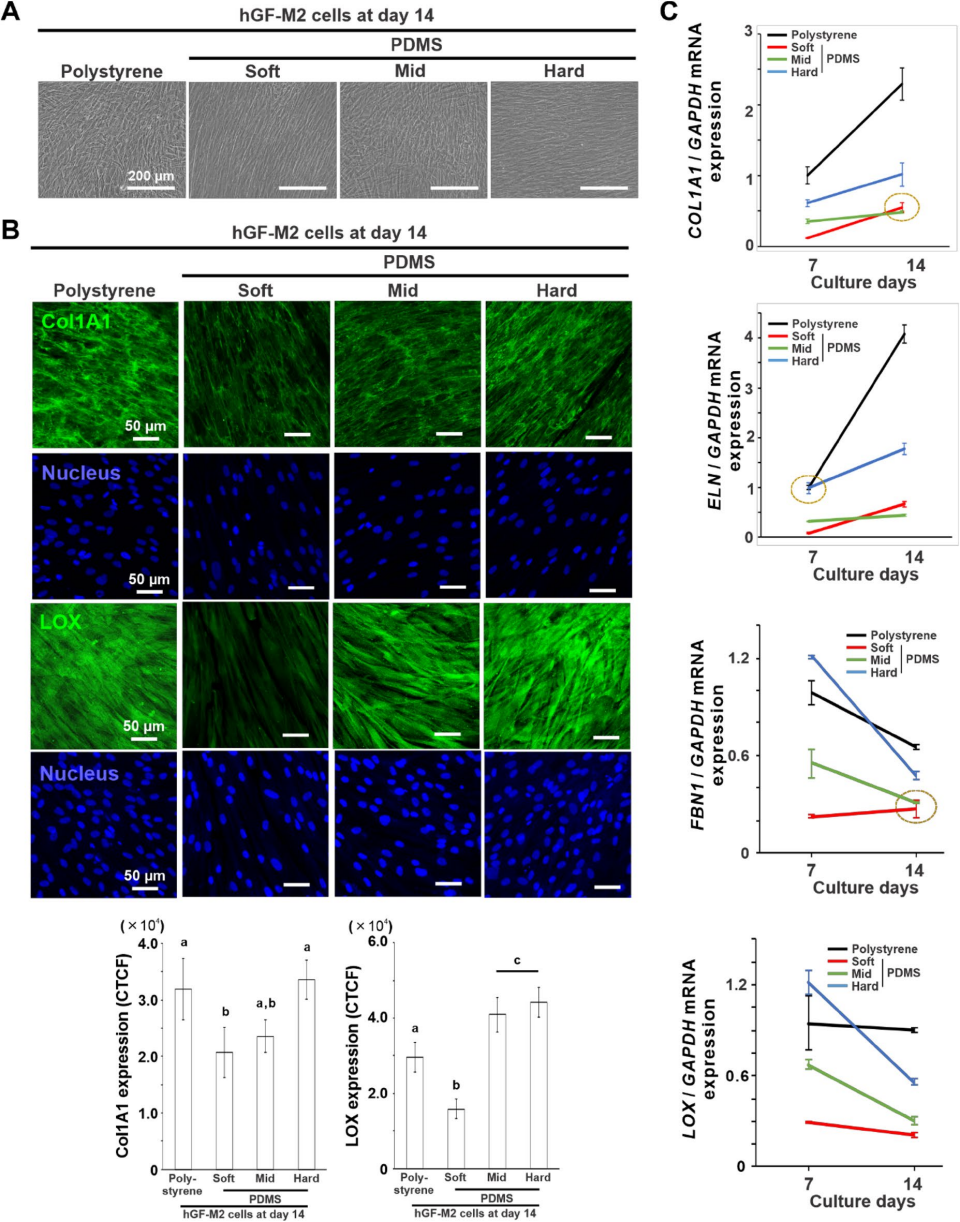
Effects of substrate stiffness on ECM production of hGFs. (**A**) Representative phase microscopic images and (**B**) immunofluorescence confocal laser microscopic images for Col1A1 or LOX (green) and nucleus (blue) and the quantifications for Col1A1 and LOX expressions based on the images in hGF-M2 cells cultured on the 0.1wt% collagen-coated polystyrene culture plate and soft, mid, and hard PDMS at days 7 and/or 14. (**C**) Reverse transcription-polymerase chain reaction (RT-PCR)-based gene expression analysis of *COL1A1*, *ELN*, *FBN1*, and *LOX* relative to *GAPDH* in hGF-M2 cells cultured under the corresponding culture condition as above on days 7 and 14. Data are represented as the means ± standard deviation (SD; *N* = 4 in B, *N* = 3 in C). Different letters indicate the statistically significant difference between them (*P* < 0.05; Tukey’s honestly significant difference [HSD] test). Yellow dotted circle indicates that there is no statistically significant difference between them (*P* > 0.05; Tukey’s HSD test). *hGFs* human gingival fibroblasts, *PDMS* polydimethylsiloxane, *Col1A1* Collagen type 1 alpha 1, *ELN* elastin, *FBN1* Fibrillin 1, *LOX* lysyl oxidase, *GAPDH* glyceraldehyde-3-phosphate dehydrogenase, *CTCF* corrected total cell fluorescence.

hGF-M2 cells remained adherent to the same extent on all types of substrates, even on day 14 (Fig. 3A). However, the expression of collagen type 1 alpha 1 (Col1A1) and lysyl oxidase (LOX) was significantly reduced on the soft PDMS as compared to that on the other substrates, particularly on the polystyrene and hard PDMS (Fig. 3B) (*P* < 0.05, Tukey’s HSD test). The expression levels of gingival ECM-related gene markers, such as *COL1A1*, elastin (*ELN)*, fibrillin 1 (*FBN1),* and *LOX*, were consistently lower on the soft PDMS than on the polystyrene and the hard PDMS both on days 7 and 14 (Fig. 3C) (*P* < 0.05, Tukey’s HSD test). The gene expression on the mid PDMS was slightly higher than that on the soft PDMS on day 7 (*P* < 0.05, Tukey’s HSD test) but became almost comparable on day 14. The day 14 hGF cultures enabled elongation along the direction axis on both the soft and hard PDMS by unidirectionally stretching PDMS substrates (Fig. S1B).

### Substrate stiffness regulates mechanotransduction and inflammatory signaling pathways in hGFs

**Figure 4:**
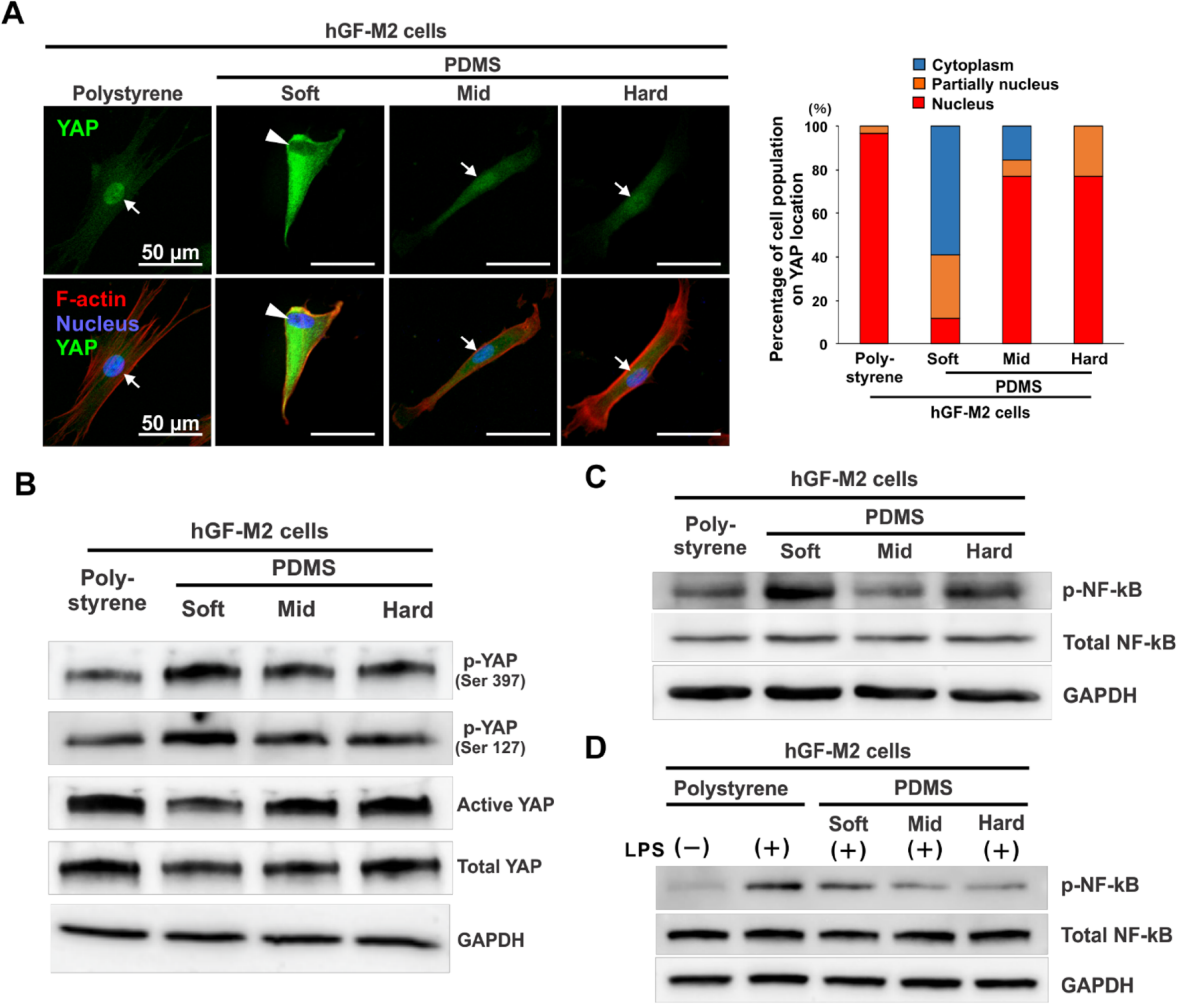
Effects of substrate stiffness on the activation of mechanotransduction and proinflammatory signaling pathways in hGFs. (**A**) Immunofluorescence confocal laser microscopic images of YAP (green), *F*-actin (red), and nucleus (blue) with a cumulative bar chart for the percentage of cell populations on YAP localization and (**B-D**) Western blotting-based expression analysis of p-YAP (Ser397), p-YAP (Ser127), active YAP, total YAP, p-NF-kB, and total NF-kB in hGF-M2 cells cultured on the 0.1wt% collagen-coated polystyrene culture plate and soft, mid, and hard PDMS for 12 h with or without co-incubation with 1,000 ng/mL of LPS. *hGFs* human gingival fibroblasts, *PDMS* polydimethylsiloxane, *LPS* lipopolysaccharide, *YAP* yes-associated protein, *p-YAP* phosphorylated Yes-associated protein, *NF-kB* nuclear factor kappa-light-chain-enhancer of activated B, *p-NF-kB* phosphorylated nuclear factor kappa-light-chain-enhancer of activated B, *GAPDH* glyceraldehyde-3-phosphate dehydrogenase.

Immunofluorescence staining analysis showed that localization of YAP signals in the nucleus was observed in hGF-M2 cells cultured for 12 h on polystyrene and mid and hard PDMS (Fig. 4A, arrows). In contrast, YAP signals in the cells on the soft PDMS were low in the nucleus but high in the cytoplasm (Fig. 4A, triangles). The percentage of cell population with distinct nuclear localization of YAP was approximately 10% on the soft PDMS in contrast to over 80% on the other substrates (Fig. 4A). Western blotting analysis demonstrated that the soft PDMS enhanced the expression of phosphorylated YAP (p-YAP [Ser397]) and p-YAP (Ser127), but reduced the expression of active and total YAP, in contrast with the opposite patterns in YAP expression on polystyrene and other PDMSs (Fig. 4B).

The expression of phosphorylated Nuclear factor kappa B (p-NF-κB) was apparently enhanced in the hGF-M2 cells on the soft PDMS as compared to that on the polystyrene or the other PDMS substrates, despite no difference in the expression of total NF-kB (Fig. 4C). Total NF-kB expression in the hGF-M2 cells did not increase on any substrates, even after co-incubation with LPS (Fig. 4D). However, p-NF-κB expression in cells after co-incubation with LPS was markedly enhanced on polystyrene and soft PDMS.

**Figure 5:**
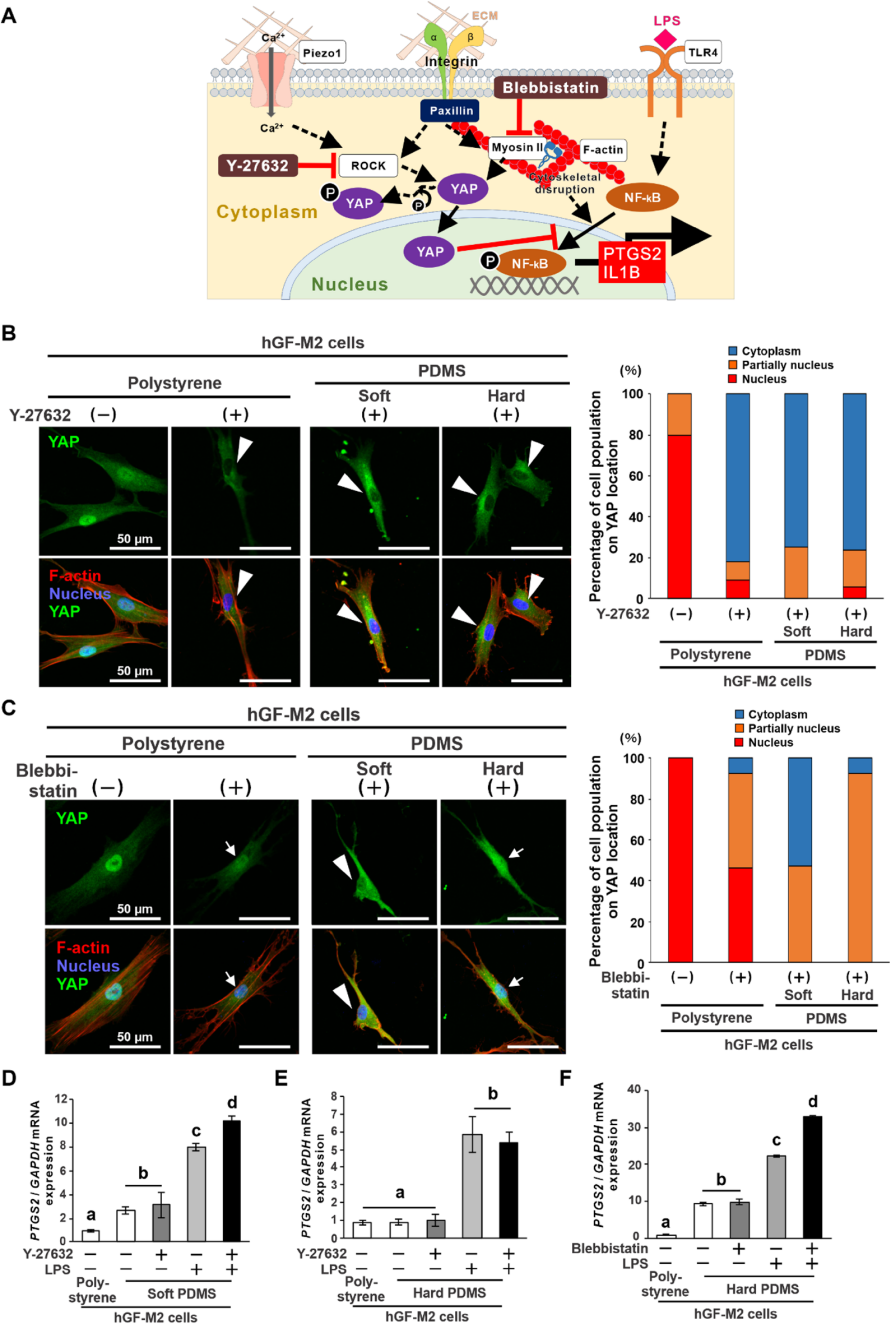
Effects of mechanotransduction inhibitors on substrate stiffness-mediated proinflammatory responses of hGFs. (**A**) Scheme showing working points of a ROCK inhibitor, Y-27632, and a myosin II inhibitor, blebbistatin, toward the blockade of YAP nuclear localization in the signaling pathway process associated with YAP and NF-kB. Immunofluorescence confocal laser microscopy images of YAP (green), *F*-actin (red), and nucleus (blue) with a cumulative bar chart for the percentage of cell populations on YAP localization in hGF-M2 cells cultured on the 0.1wt% collagen-coated polystyrene culture plate and soft and hard PDMS for a total of 12 h via 2-h co-incubation with or without 5 µM Y-27632 (**B**) or 30 µM blebbistatin (**C**). Reverse transcription-polymerase chain reaction (RT-PCR)-based gene expression analysis of *PTGS2* relative to GAPDH in hGF-M2 cells cultured on the 0.1wt% collagen-coated polystyrene culture plate and the soft (**D**) or hard PDMS (**E** and **F**) for a total of 24 h, including 2 h of co-culture with 5 µM Y-27632 (**D** and **E**) or 30 µM blebbistatin (**F**) followed by 12 h of co-culture with LPS. Data are represented as the means ± standard deviation (SD; *N* = 3). Different letters indicate the statistically significant difference between them (*P* < 0.05; Tukey’s honestly significant difference [HSD] test). *hGFs* human gingival fibroblasts, *PDMS* polydimethylsiloxane, *PTGS2* prostaglandin G/H synthase 2, *IL1B* Interleukin-1β, *ROCK* rho-associated, coiled-coil containing protein kinase, *LPS* lipopolysaccharide, *YAP* yes-associated protein, *NF-kB* nuclear factor kappa-light-chain-enhancer of activated B, *GAPDH* glyceraldehyde-3-phosphate dehydrogenase. Note that similar to the soft PDMS, co-incubation with Y-27632 suppressed YAP signals in the cell nucleus (**B**, tringles) on all types of substrates, but co-incubation with blebbistatin reduced YAP signals in the cell nucleus on polystyrene and hard PDMS (**C**, arrows), in contrast with no detection of YAP-labeled nucleus on soft PDMS (**C**, triangles).

A Rho-associated protein kinase (ROCK) inhibitor, Y-27632 (Fig. 5A), reduced the YAP-labelling in the hGF-M2 cell nucleus down to approximately 20% of the cell population on all types of substrates (Fig. 5B), whereas a myosin II inhibitor (Fig. 5A), blebbistatin, kept the YAP-labelling within the nucleus in approximately 80% of the cell population on polystyrene and hard PDMS (Fig. 5C). The Y-27632 ROCK inhibitor augmented the LPS-induced upregulation of *PTGS2* gene expression in the hGF-M2 cells on the soft PDMS (Fig. 5D) (*P* < 0.05, Tukey’s HSD test), but not in the hard PDMS (Fig. 5E) (*P* > 0.05, Tukey’s HSD test). In contrast, co-incubation with a blebbistatin myosin II inhibitor augmented the LPS-induced upregulation of *PTGS2* gene expression in the hGF-M2 cells on the hard PDMS (Fig. 5F) (*P* < 0.05, Tukey’s HSD test).

### Substrate stiffness regulates formation of focal adhesion in hGFs

**Figure 6:**
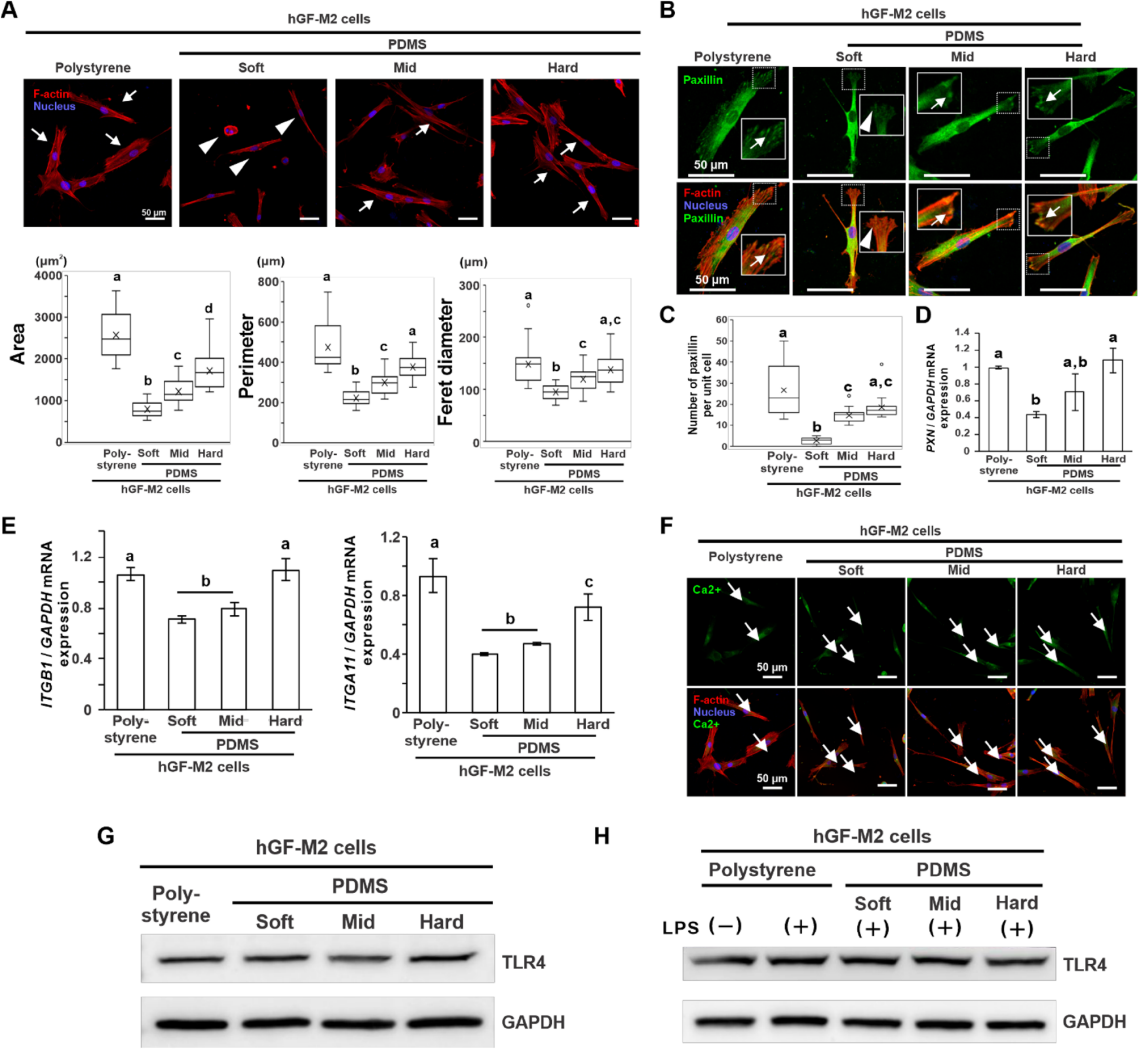
Effects of substrate stiffness on the cell morphology and focal adhesion in hGFs. (**A**) Representative confocal laser microscopic images of *F*-actin (red) and nucleus (blue) and the corresponding cytomorphometry parameters regarding area, perimeter, and Feret diameter in hGF-M2 cells cultured on the 0.1wt% collagen-coated polystyrene culture plate and soft, mid, and hard PDMS for 12 h. Immunofluorescence confocal laser microscopic images of paxillin (**B**) or intracellular calcium ions (**F**) (green), *F*-actin cytoskeleton (red), and nucleus (blue), (**C**) the number of paxillin per unit cell analyzed on the corresponding immunofluorescence confocal microscopy images, and Reverse transcription-polymerase chain reaction (RT-PCR)-based gene expression analysis of *PXN* (**D**), *ITGB1* and *ITGA11* (**E**) relative to *GAPDH* in hGF-M2 cells cultured on the 0.1wt% collagen-coated polystyrene culture plate and soft, mid, and hard PDMS for 12 h. Data are represented as the means ± standard deviation (SD; *N* = 15 in **A** and **C**, *N* = 3 in **D** and** E**). (**G**,**H**) Western blotting analysis of the TLR4 and GAPDH protein expression in hGF-M2 cells cultured on the 0.1wt% collagen-coated polystyrene culture plate and soft, mid, and hard PDMS without and with co-incubation with 1,000 ng/mL of LPS for 12 h. Different letters indicate the statistically significant difference between them (*P* < 0.05; Dann-Bonferroni test in (**A**) and (**C**), Tukey’s honestly significant difference [HSD] test in **D**,**E**). Squared images in (**B**) indicate the expanded images corresponding to the area surrounded by dashed squares. Note: (**A**) Small or rounded shapes with less cytoskeletal development on the soft PDMS (triangles) in contrast with well-spread spindle shapes with cytoskeletal development on the other substrates (arrows), (**B**) less paxillin signals on the tips of cellular projections (triangles) of hGF-M2 cells on soft PDMS in contrast with many signals (arrows) in the cells on the polystyrene and the other PDMS substrates, and (**F**) the green signals (arrows) are visible in hGF-M2 cells on all types of substrates. *hGFs* human gingival fibroblasts, *PDMS* polydimethylsiloxane, *ECM* extracellular matrix, *PXN* paxillin, *ITGB1* Integrin $\beta$1, *ITGA11* Integrin $\alpha$11, *Ca*^*2+*^ calcium ions, *LPS* lipopolysaccharide, *TLR4* toll-like receptor 4, *GAPDH* glyceraldehyde-3-phosphate dehydrogenase.

The hGF-M2 cells on the soft PDMS showed relatively small spindles and rounded shapes with less cytoskeletal development (Fig. 6A, triangles) in contrast to the more spread and rectangular cell shapes with more organized cytoskeletons on the other substrates (Fig. 6A, arrows). The area, perimeter, and Feret diameter were the lowest on the soft PDMS (Fig. 6A) (*P* < 0.05, Dann-Bonferroni test) and increased in the cells with increasing PDMS stiffness up to levels equivalent to those on polystyrene (*P* > 0.05).

The expression of the focal adhesion adaptor protein, paxillin (PXN), on the cellular projections was hardly detected in the hGF-M2 cells on the soft PDMS (Fig. 6B, triangles) in contrast with the apparent signal detection in the cells on the other substrates (Fig. 6B, arrows). The number of paxillin per unit cell and the expression of *PXN* were the lowest on soft PDMS (Fig. 6C,D) (*P* < 0.05, Tukey’s HSD test). These values on PDMS gradually increased with increasing PDMS stiffness up to levels equivalent to those on polystyrene (*P* > 0.05, Tukey’s HSD test). In addition, the expression levels of integrin-related genes, such as integrin $\beta$ 1 *(ITGB1)* and integrin $\alpha$ 11 *(ITGA11)*, in hGF-M2 cells were the lowest on the soft PDMS (Fig. 6E) (*P* < 0.05, Tukey’s HSD test) but were comparable on the hard PDMS with those on the polystyrene.

Intracellular calcium ions, indicating activation of mechano-sensing molecules Piezo1, were observed in the hGF-M2 cells on any type of substrate (Fig. 6F). Expression of a representative LPS receptor, Toll-like receptor 4 (TLR4), was not changed in the hGF-M2 cells cultured on any type of substrate, regardless of LPS co-incubation (Fig. 6G,H).

## Discussion

The stiffness of the mid and hard PDMS substrates was 17.0 and 26.2 kPa on average, respectively, which was comparable to or more than approximately 20 kPa of healthy gingival tissue^22,23^. PDMS has been used as a culture substrate that mimics the elastic modulus of living tissues^24,25^ and is lower in hysteresis than hydrogel materials such as polyacrylamide^26^, so that it can avoid undesirable strain accumulation as much as possible. PDMS substrates are assumed to show isotropic elasticity because the substrate is flat without any topographical surface patterning. Furthermore, an ECM coating is required on the PDMS^27^ due to poor cell adhesion properties as seen in this study (Fig. S1A). The native collagens used herein retain the inherent type I collagen fibril structures, such as a triple helix structure and C- and N-terminal telopeptides, but do not retain cell-derived bioactive substances, nucleic acids, or MMPs. The cytocompatibility is widely accepted in biomaterial science^28,29^. The hGFs were stretched along the direction axis of the PDMS substrates at day 14, regardless of the stiffness (Fig. S1B). This indicated that the cells strongly adhere to the PDMS substrates via the coated collagen and/or the self-produced ECM during the culture periods. Similar to how an artificial elastic material coated with adhesive proteins can be used for investigating the relationship between the ECM stiffness and cellular function^28,30^, the collagen-coated PDMS substrates provided an acceptable culture environment for investigating the cellular mechano-response to substrate stiffness.

The soft PDMS substrates with 4.4 kPa stiffness induced proinflammatory responses in the primary hGFs regardless of LPS co-incubation, despite no influence on cell attachment (Fig. 1B–E). Substrate stiffness-mediated induction of proinflammatory responses in hGFs was reduced with increasing substrate stiffness. The soft PDMS substrate markedly increased the expression of proinflammatory mediators in the hGFs under inflammatory condition with LPS (Fig. 1F–H). The expression of proinflammatory mediators in hGFs was reduced with increasing substrate stiffness. These phenomena were also observed in other cell populations isolated from different individuals (Fig. S2). In addition, the culture supernatants from the hGFs culture on the soft PDMS substrate induced THP-1 cells to express the typical morphological features^31^ and biomarkers of M1 macrophages (Fig. 2). These findings suggest that gingival fibroblasts play a unique role in the progressions and prolongations of periodontal inflammation^4,32^ through the regulation of proinflammatory responses by gingival ECM stiffness.

**Figure 7:**
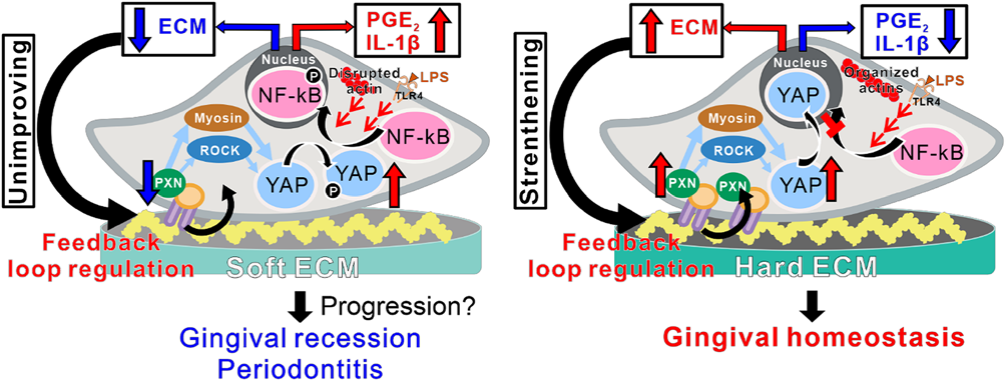
Possible cellular mechanisms underlying substrate stiffness-mediated proinflammatory responses of hGFs. A hypothetical scheme showing the cellular mechanotransduction mechanisms underlying the regulation of proinflammatory responses and ECM production in human gingival fibroblasts by ECM stiffness. The soft ECM inactivates YAP and activates NF-kB in gingival fibroblasts via disruption of focal adhesion formation and cytoskeletal development, even in the absence of LPS stimulation. Inactive YAP cannot stop nuclear translocation of p-NF-kB, leading to the expression of proinflammatory mediators, such as prostaglandin E2 (PGE_2_) and IL-1 $\beta$ and suppression of ECM synthesis in gingival fibroblasts particularly under an inflammatory condition. The unimproved ECM structure of the soft ECM continues to regulate the feedback loop of gingival fibroblasts according to these cascades, possibly progressing gingival recession and periodontitis (left). On the other hand, the hard ECM prevents proinflammatory responses and strengthens ECM structure via inactivation of NF-kB by active YAP translocated into nucleus (right). The rigid structure of ECM keeps gingival homeostasis by feedback loop regulation of gingival fibroblasts with physical signals of ECM stiffness (right). *hGFs* human gingival fibroblasts, *ECM* extracellular matrix, *PXN* paxillin, *ROCK* rho-associated, coiled-coil containing protein kinase, *LPS* lipopolysaccharide, *TLR4* toll-like receptor 4, *YAP* yes-associated protein, *NF-kB* nuclear factor kappa-light-chain-enhancer of activated B, *PGE*_*2*_ prostaglandin E2, *IL-1β* Interleukin-1β.

The soft PDMS substrate inhibited the production of ECM and cross-linking agent in the hGFs without loss of cell attachment (Fig. 3). The mid and soft PDMS substrates consistently downregulated gingival ECM-related gene expression, whereas hard PDMS supported high expression of these characteristics to the levels on polystyrene. ECM stiffness is associated with the progression of various fibrotic tissues, such as progressive lung fibrosis^33^, intestinal fibrosis^34^, and dermal fibrosis^35^, by mediating the repeated cycle of proinflammatory responses and ECM production in various types of fibroblasts. Gingival fibroblasts might have tried to form an ECM structure matching the mechanical properties of the surrounding microenvironment by regulating their ECM production capability, to make the hard substrates becomes harder and the mid or soft substrates remain the same. More importantly, the ECM synthesis capability of hGFs is sensitively regulated by substrate stiffness, together with proinflammatory responses. Substrate stiffness might exert feedback loop regulation of proinflammatory responses and ECM production in gingival fibroblasts to maintain homeostasis of the harder gingiva or lead to gradual degradation of the softer gingiva (Fig. 7).

Inhibition of YAP nuclear localization was observed in the hGFs on the soft PDMS substrates, in contrast to the opposite reactions on the mid or hard PDMS or polystyrene substrates (Fig. 4A,B). These observations indicated that the soft PDMS substrate inhibited cellular mechanotransduction in gingival fibroblasts, in contrast with no change in YAP behavior on mid and hard PDMS. In addition, soft PDMS increased p-NF-kB expression, whereas mid and hard PDMS did not, regardless of the inflammatory condition (Fig. 4C,D). Gingival fibroblasts produce proinflammatory mediators via the NF-kB pathway under inflammatory condition^36^. Active YAP may inhibit NF-kB activation inside and outside the nucleus, resulting in the inhibition of proinflammatory responses in various cell types, such as macrophages^18^ and chondrocytes^37^. Taken together, the soft substrate might have not interfered with the activation of NF-kB by inactivating YAP, leading to proinflammatory responses and ECM synthesis inhibition in the hGFs regardless of inflammatory condition, whereas the hard substrate might have induced the opposite signaling cascade to prevent these biologically adverse reactions (Fig. 7).

Despite successful inhibition of YAP nuclear localization (Fig. 5B), a ROCK inhibitor, Y-27632, did not further increase LPS-mediated proinflammatory responses in the hGFs on the hard PDMS (Fig. 5E), as opposed to the exacerbation on the soft PDMS (Fig. 5D). On the other hand, although only YAP nuclear localization was reduced in the hGFs (Fig. 5C), a myosin II inhibitor, blebbistatin, further increased LPS-mediated proinflammatory responses in the hGFs on the hard PDMS substrate (Fig. 5F). These findings indicate that YAP behavior does not govern all of the inflammatory responses and ECM production capability of hGFs by substrate stiffness. In addition, the hGFs became short and small cell shapes with less cytoskeletal development on the soft PDMS, whereas the cell length and development of cellular projections and cytoskeleton gradually recovered with increasing substrate stiffness (Fig. 6A). Actomyosin and cytoskeletal arrangements are closely associated with the progression of cellular mechanotransduction^38^. Actin cytoskeletal disruption induces cellular proinflammatory responses through NF-kB activation^39,40^. Focal adhesion plaques function as mechano-sensing to activate YAP via activation of the ROCK-myosin signaling pathway^41^. The expression of paxillin in the hGFs increased almost with increasing PDMS stiffness at both the gene and protein levels (Fig. 6B–D). Integrin gene expression in hGFs was also upregulated on harder substrates than on softer substrates (Fig. 6E). Other possibilities, such as other mechano-sensing molecule calcium ion channels, Peizo 1^42^, or the mechano-responsive proinflammatory ligand, TLR4^43^ were not involved in the soft substrate-induced proinflammatory responses in the hGFs (Fig. 6F-H). Taken together, the soft substrate-induced proinflammatory responses in hGFs might be attributed to the inhibition of focal adhesion formation and cytoskeletal development leading to NK-kB activation (Fig. 7).

This study had limitations in elucidating the relationship between gingival ECM stiffness and inflammation of gingival tissue. Although gingival fibroblasts are the dominant cell population, gingival tissue consists of the heterogeneous cell populations including tissue forming, immune and stem cells. For instance, proinflammatory responses of macrophages is turned by substrate stiffness via YAP signaling pathway^18^. Proinflammatory responses of the gingival resident immune cells to substrate stiffness is particularly of interest for the future research. In addition, influences of gingival ECM stiffness on expressions of anti-inflammatory and tissue inhibitor metalloproteinases, fibroblast-to-myofibroblast transition and the detailed interaction with the innate immunity^44^ should be further investigated in gingival fibroblasts. Furthermore, the PDMS two-dimensional culture model used in this study is not ideal as a biomimetic microenvironment. To prove that gingival inflammation mediated by mechanotransduction of gingival fibroblasts, comprehensive biochemical and functional analyses are required in a three-dimensional culture model or an animal model regulating gingival stiffness without an inflammation. However, For the first time, this study proposed the possible relationship between gingival ECM stiffness and gingival fibroblastic proinflammatory response and ECM production capability. The phenomenon might underlie the high susceptibility for gingival recession in patients with the poor gingival phenotype^45,46^. The concept of ECM stiffness-mediated regulation of proinflammatory reactions in gingival fibroblasts not only supports the clinical significance of the gingival phenotype but also provides important information for the fields of oral immunology and matrix biology.

## Material and methods

### Preparation of culture substrates mimicking gingival tissue stiffness

A vinyl-terminated base and methyl hydrogen siloxane curing agent of commercial PDMS (Sylgard 527; Dow Corning, NY, USA) were mixed at 5:4, 1:1, and 4:5 weight ratios as soft, mid, and hard PDMS, respectively. This mixture was polymerized directly on the bottom of a tissue culture plate at 65 °C for 4 h under the atmosphere after defoaming at − 1 Pa for 3 h. The Young's modulus of each PDMS substrate was measured using a creep meter (RE2-33005C; YAMADEN, Tokyo, Japan). Polystyrene culture plates or sterilized PDMS substrates were coated with 0.1wt% bovine dermis-derived native type I collagen solution (IAC-30; Koken Co., Ltd., Tokyo, Japan). The plates were then incubated at room temperature for 90 min.

### hGF culture

Two populations of hGFs isolated from two different healthy donors were used in a previous study^47^ which had been approved for human gingival tissue collection, subsequent genetic modification, genome/gene analyses, and the secondary use of cell sources by the Institutional Review Board at Osaka University Graduate School of Dentistry (approval number: H21-E7) and the Ethics Committee for Human Genome/Gene Analysis Research at Osaka University (approval number: 233). These two populations of hGFs were labeled hGF-M2 and hGF-F1. The hGFs were grown in the hGF growth medium consisting of Dulbecco's minimal essential medium/Ham's F12 (DMEM/F12) supplemented with 10% fetal bovine serum (FBS; Japan Bioserum), 100 U of penicillin, and 100 μg/mL of streptomycin (FUJIFILM Wako Pure Chemical Corporation) at 37 °C in a 5% CO_2_ atmosphere and underwent 5–9 passages. After 80% confluence, the cells were detached with 0.25% trypsin/1 mM ethylenediaminetetraacetic acid and seeded in the hGF growth media on collagen-coated substrates of a 12- or 24-well culture-graded polystyrene plate or the soft, mid, or hard PDMS at 8.0 × 10^3^ or 1.0 × 10^5^ cells/cm^2^. The cells were cultured for up to 14 days at 37 °C in a 5% CO_2_ atmosphere, and the medium was renewed every three days.

The culture supernatants of hGF-M2 cells cultured for 24 h on each substrate were collected and filtered through a Millex-Gp 0.22 µm membrane filter (Merck Millipore Ltd., Carrigtwohill, Ireland) into a tube. The supernatants were stored at –80 ℃ prior to use in the subsequent monocyte experiment.

### Induction of inflammatory response and inhibition of cellular mechanotransduction

To evaluate the influence of substrate stiffness on further proinflammatory responses of hGFs under inflammatory condition, LPS extracted from *Escherichia coli* O55:B5 (Sigma-Aldrich, St. Louis, MO, USA) was used. The cell cultures were added at a final concentration of 0, 10, 100, or 1000 ng/mL LPS at cell seeding or with medium change after 2 h of co-incubation with the following the cellular mechanotransduction inhibitor. The cells were co-incubated with LPS for 12 h at 37 °C in a 5% CO_2_ atmosphere and then evaluated for proinflammatory responses.

To investigate the cellular signaling pathways involved in the induction of proinflammatory responses in gingival fibroblasts by substrate stiffness, two inhibitors with different points of action on cellular mechanotransduction were used. Y-27632 (CultureSure®Y-27632, FUJIFILM Wako Pure Chemical Corporation) and blebbistatin (B592500, Toronto Research Chemicals, Toronto, ON, Canada) inhibited ROCK and myosin II, respectively. After 10-h incubation, the hGF culture on the collagen-coated polystyrene culture plate or soft or hard PDMS was coincubated with 0, 5, or 10 μM Y-27632 or 0, 10, 30, or 50 μM blebbistatin for 2 h at 37 °C in a 5% CO_2_ atmosphere. After 2-h of co-incubation with the inhibitor, hGF cultures were subjected to LPS co-incubation as described above.

### Human monocyte cell line culture

The human monocytic leukemia cell line, THP-1 (JCRB0112.1; Japanese Collection of Research Bioresources Cell Bank, Osaka, Japan), was co-cultured with the supernatants from the hGFs cultured on each substrate. The cells were grown in THP-1 growth medium consisting of Roswell Park Memorial Institute 1640 basal medium supplemented with 0.03% l-glutamine, 10% FBS, 10 mM HEPES, 1 mM sodium pyruvate (Sigma-Aldrich), 100 U of penicillin, and 100 μg/mL of streptomycin solution in a humidified 5% CO_2_ atmosphere. At 80% confluence, the cells were seeded on a 24-well culture-graded polystyrene plate at 8.0 × 10^3^ or 1.0 × 10^5^ cells/cm^2^ and cultured in the macrophage differentiation medium consisting of the THP-1 growth medium supplemented with 100 nM phorbol 12-myristate-13-acetate (PMA) at 37 °C in a 5% CO_2_ atmosphere. After 24-h of incubation, the culture was replaced with fresh macrophage differentiation medium with or without the reagents for M1 or M2 induction as control conditions or DMEM/F12 supernatants from hGF-M2 cells cultured on each substrate according to previously reported protocols^48,49^. Fresh DMEM/F12 basal medium or hGF culture supernatants were mixed with the macrophage differentiation medium in a 1:1 volume ratio. At that time, both the final concentration of L-glutamine and PMA and the working medium volume per well were adjusted to be the same as the M1 and M2-induced control cultures. After renewing each culture medium, the THP-1 cells were cultured for additional 24 h at 37 °C in a 5% CO_2_ atmosphere and then evaluated for cell morphology and gene expression of M1 polarization markers.

### Cell appearance and attachment assay

Cell appearances of hGFs and THP-1 cells on polystyrene and/or PDMS substrates were observed under a phase contrast microscope. Cell morphometries were performed on microscopic images of THP-1 cells using ImageJ software version 1.53t (National Institutes of Health, Bethesda, MD, USA) to evaluate aspect ratio and circularity.

The cytocompatibility of each PDMS substrate was evaluated via phase microscopy and water-soluble tetrazolium (WST)-1-based assay (Roche Diagnostics, Tokyo, Japan) for quantification of adherent cells in the hGF culture. For WST-1-based colorimetry, a 10% v/v WST-1 reagent was added to the culture medium. The culture plate was incubated at 37 °C for 3 h and then the supernatants were transferred into a 96-well microplate. The amount of formazan produced in the supernatant was measured using an enzyme-linked immunosorbent assay reader at a wavelength of 450 nm.

Methylene blue staining was used to visualize the attached hGFs on the collagen-coated polystyrene culture plate and PDMS, 12 h after seeding. The cells were fixed with 10% buffered formalin (FUJIFILM Wako Pure Chemical Corporation) and stained with 1.4% methylene blue solution for 30 min at room temperature. After washing with 10 mM sodium borate buffer, stained cells were observed under a light microscope.

### Reverse transcription-polymerase chain reaction (RT-PCR)

The total RNA in the culture was extracted using TRIzol reagent (Ambion/Life Technologies, Carlsbad, CA, USA) on PDMS and polystyrene plates. RNA isolation and purification were performed using an RNAeasy® Mini Kit (Qiagen, Hilden, Germany), followed by DNase treatment and removal (Thermo Fisher Scientific, Waltham, MA, USA). Complementary DNA (cDNA) was synthesized using a PrimeScriptTM II 1st Strand cDNA Synthesis Kit (Takara Bio, Shiga, Japan). Messenger RNA (mRNA) expression was determined using real-time RT-PCR. mRNA expression was determined using the StepOnePlus Real-Time PCR system (Applied Biosystems, Thermo Fisher Scientific) and Thunderbird® SYBR® qPCR Mix (Toyobo, Osaka, Japan) for the SYBR-green-based PCR reaction. The target gene expression levels were quantitatively analyzed using the comparative cycle time (ΔΔCT) method. GAPDH was used as the housekeeping gene. The primers used are listed in Supplementary Table S1.

### Quantification of PGE_2_

PGE_2_ concentration in the culture supernatant was quantified using a competitive immune assay with high sensitivity (ADI-900-001; Enzo Life Sciences, Farmingdale, NY, USA). The assay is based on a competitive reaction to an anti-PGE_2_ monoclonal antibody between alkaline phosphatase covalently bound to PGE_2_ and the sample. Briefly, the samples or PGE_2_ standards were co-incubated with an anti-PGE_2_ monoclonal antibody and alkaline phosphatase covalently bound to PGE_2_ in a 96-well GxM IgG microtiter plate for 2 h at room temperature on a shaker. After washing with unbound antibody and PGE_2_, the plates were reacted with p-nitrophenylphosphate for 45 min at room temperature to develop a color against alkaline phosphatase. The optical density of the plate was read at 405 nm using a microplate reader. The PGE_2_ concentration was quantified against a standard curve.

After collecting the culture supernatants for PGE_2_ quantification, the total DNA in the adherent cells was measured using a DNA quantification kit (COSMO BIO Co., Ltd., Tokyo, Japan) according to the manufacturer's instructions. After washing with phosphate-buffered saline (PBS), the cells were lysed with − 80 °C freezing and thawing cycles in a cell lysis buffer. The cell lysate was mixed with an equal volume of Hoechst 33258 and diluted 20-fold with the same cell lysis buffer. The mixture was then transferred to 96-well black and flat-bottom microplates. The fluorescence intensity was measured using a GloMax-Multi Detection System reader (Promega Corporation, Madison, WI, USA) (excitation at 365 nm and emission at 410–460 nm).

The concentration of PGE_2_ was normalized to the DNA concentration of the adherent cells on each substrate and expressed as the PGE_2_ level per unit DNA in adherent cells.

### Immunofluorescence staining and analyses

The cells on the collagen-coated polystyrene culture plate and PDMS were fixed with a 4% paraformaldehyde phosphate buffer solution (FUJIFILM Wako Pure Chemical Corporation) for 15 min. After washing with PBS, the cells were blocked for non-specific protein binding using a blocking buffer containing 3.0% bovine serum albumin (BSA) (FUJIFILM Wako Pure Chemical Corporation), 0.1% Triton-X (FUJIFILM Wako Pure Chemical Corporation), and 0.01% Tween 20 (Sigma-Aldrich) for 60 min. In the cases to detect the following specific cellular markers, the cells were fixed, permeabilized, and blocked for non-specific proteins, were incubated in the primary antibodies such as 1/200 anti- inducible nitric oxide synthase (ab3523, Abcam, Cambridge, UK), 1/200 anti-YAP (sc-101199, Santa Cruz Biotechnology, Dallas, TX, USA), 1/200 anti-LOX (sc373995, Santa Cruz Biotechnology), 1/200 anti-COL1A1 (NBP1-30054, Novus Biologicals, Centennial, CO, USA), and 1/250 anti-paxillin (ab32084, Abcam) overnight at 4 ℃. After washing with PBS, the cells were incubated with the secondary antibody Alexa Fluor 488 (H&L) together with 1/500 Hoechst 33258 pentahydrate (bis-benzidamine) (Thermo Fisher Scientific) for nuclear staining and 1/500 rhodamine phalloidin (Thermo Fisher Scientific) for *F*-actin staining for 90 min at room temperature in the dark. When only cell morphology or numbers of adherent cells were evaluated, only F-actin and/or nuclear staining without antibody treatment was performed after cell fixation. The PDMS substrate of the day 14 culture was stretched after cell fixation to confirm persistent cell attachment to the substrate throughout the culture period. For that purpose, F-actin and nuclear staining were performed with the PDMS substrates that were stretched along a unidirectional axis at an elongation rate of 9%. The cells were washed with PBS and mounted on a glass-bottomed dish (Matsunami Glass Ind., Ltd., Osaka, Japan) with 90% glycerol. Cells were observed under an LSM 780 confocal laser microscope (Carl Zeiss, Jena, Germany). Measurements of the arbitrary unit areas of COL1A1 and LOX signals, the nuclear numbers in randomly selected areas with 0.09 mm^2^, percentages of cell populations on nuclear or cytoplasmic localization of YAP, and cell morphometries for cell area, perimeter, Feret diameter, and number of paxillin molecules per unit cell were performed on confocal laser microscopic images using ImageJ software. COL1A1 and LOX expressions were evaluated as corrected total cell fluorescence (CTCF) by calculating the signal area per nuclear unit. Localization of YAP signals was categorized into “nucleus” or “cytoplasm” based on the biased or uniform distribution of the fluorescent signal in each region. Furthermore, YAP signals almost evenly distributed in the nucleus and cytoplasm were categorized as “partially nucleus.” The percentages of cells belonging to each category to the total number of cells was calculated for each substrate. Paxillin expression was measured only at the tip of the cell projection.

### Western blotting analysis

The cells on the collagen-coated polystyrene culture plate and PDMS were scraped and lysed by ultrasonication in radioimmunoprecipitation assay (RIPA) buffer (FUJIFILM Wako Pure Chemical Corporation) containing protease and phosphatase inhibitors. Cell lysates were centrifuged at 15,000×*g* for 20 min. The supernatants were mixed with 2-mercaptoethanol and 4 × Laemmli sample buffer and incubated at 95 °C for 5 min. After mixing the sample with an ionic detergent (Thermo Fisher Scientific), the total protein concentration of each sample was measured using a protein colorimetric assay (Pierce 660 nm Protein Assay, Thermo Fisher Scientific). The protein samples were loaded onto a 12% polyacrylamide gel (SDS-PAGE) for electrophoresis at 120 V for 90 min, followed by transfer to a polyvinylidene difluoride (PVDF) membrane (Immun-Blot PVDF Membrane, Bio-Rad Laboratories, Inc., Hercules, CA, USA). After blocking with 5% skim milk (Becton Dickinson, Franklin Lakes, NJ, USA) in tris-buffered saline with 0.1% Tween 20 (TBS-T) for 1 h at room temperature, the samples were reacted with the primary antibody such as 1/1000 anti-serine 397-phosphorylated YAP [p-YAP (Ser397)] (D1E7Y, Cell signaling Technology, Danvers, MA, USA), 1/1000 anti-serine 127-phosphorylated YAP [p-YAP (Ser127)] (D9W2I, Cell signaling Technology), 1/1000 anti-active YAP (ab205270, Abcam), 1/1000 anti-YAP (total YAP) (sc-101199, Santa Cruz Biotechnology), 1/1000 anti-NF-κB p65 (sc-8008, Santa Cruz Biotechnology), 1/800 anti-serine 536- p-NF-κB p65 (sc-136548, Santa Cruz Biotechnology), 1/1000 anti-TLR4 (sc-293072, Santa Cruz Biotechnology) and 1/2500 anti-GAPDH (MAB374, Millipore) overnight at 4 °C. The membrane was rinsed with TBS-T and then incubated with 1/2000 horse radish peroxidase (HPR)-labeled anti-mouse IgG kappa binding (sc-516102, Santa Cruz Biotechnology) or 1/2000 HPR-labeled anti-rabbit IgG (sc-2357, Santa Cruz Biotechnology) monoclonal antibody for 1 h at room temperature. Signals were visualized with enhanced chemiluminescence HRP substrate (SuperSignal™, Thermo Fisher Scientific) using an image analyzer (ImageQuant LAS-500, GE Healthcare, Chicago, IL, USA).

### Detection of intracellular calcium ions

The hGF-M2 cells were cultured on collagen-coated polystyrene and PDMS substrates for 12 h and then co-incubated with 1 μM 1-[2-Amino-5-(2,7-difluoro-6-acetoxymethoxy-3-oxo-9-xanthenyl)phenoxy]-2-(2-amino-5-methylphenoxy)ethane-N,N,N′,N′-tetraacetic acid, Ftetra(acetoxymethyl) ester (Fluo 4-AM, Dojindo) for 1 h in order to detect intracellular calcium ions. Next, the cells were fixed in a 4% paraformaldehyde phosphate buffer solution for 10 min. After rinsing thrice with PBS, the cells were incubated in 1/500 Hoechst for nuclei and 1/500 rhodamine phalloidin for cytoskeletons in blocking buffer with 5% BSA, 0.1% Triton X, and 0.01% Tween for 90 min. After rinsing with PBS three times, the cells were mounted on a glass-bottomed dish with 90% glycerol and observed using a confocal laser microscope.

### Statistical analysis

For the PDMS stiffness test, three independent samples from each group were analyzed. All the culture experiments were performed in at least three independent cell batches on multiple days (*N* = 3). Cell morphometries were analyzed in single cell images in randomly selected phase-contrast and confocal laser microscopic images in multiple cultures (*N* = 11–31 for YAP localization, *N* = 20 for cell shapes of THP-1 cells, *N* = 15 for cell shapes of hGFs and paxillin expression). Nuclear numbers and COL1A1 and LOX expressions were analyzed in four randomly selected confocal laser microscopic images of multiple cultures (*N* = 4). One-way analysis of variance (ANOVA) or Kruskal–Wallis H-test was used to assess differences among multiple experimental groups, while two-way ANOVA was used to assess the interactions between differences in substrate types and medium components. When appropriate, post-hoc Tukey’s honestly significant difference test or the Dann-Bonferroni test was used. *P* < 0.05 was considered a statistically significant difference. All statistical analyses were performed using IBM SPSS Statistics version 21 (IBM Japan, Ltd., Tokyo, Japan).

## Conclusion

The stiffness of ECM-coated substrates regulated the proinflammatory reaction and ECM synthesis capacity of hGFs. It induced proinflammatory responses on soft substrate while inhibiting these responses and promoting ECM production on hard substrate. Moreover, the substrate stiffness-regulated proinflammatory responses in hGFs are mediated by a signaling pathway associated with cellular mechanotransduction.

## Supplementary Information

Supplementary Information 1.

Supplementary Information 2.

## Data Availability

All raw and processed data in the present study are available from the corresponding author on reasonable request.
