# Supplementary material for: Substrate stiffness controls proinflammatory responses in human gingival fibroblasts
Source: Sci Rep. 2023 Jan 24;13:1358. doi: 10.1038/s41598-023-28541-z (PMC9873657; doi:10.1038/s41598-023-28541-z)
Supplement: Supplementary file 1 — Supplementary Information 1. [file 41598_2023_28541_MOESM1_ESM.pdf]

Sci Rep. [Original Research]

Substrate stiffness controls proinflammatory responses in human gingival fibroblasts

Watcharaphol Tiskratok[1,2], Masahiro Yamada[1]*, Jun Watanabe[1], Nadia Kartikasari[1], Tsuyoshi Kimura[3], and Hiroshi Egusa[1, 4]*

[1] *Division of Molecular and Regenerative Prosthodontics, Tohoku University Graduate School of Dentistry, Sendai, Miyagi, Japan.*

[2] *Institute of Dentistry, Suranaree University of Technology, Mueang Nakhon Ratchasima District, Nakhon Ratchasima 30000, Thailand.*

[3] *Institute of Biomaterials and Bioengineering, Tokyo Medical and Dental University, Chiyoda-ku, Tokyo, Japan*

[4] *Center for Advanced Stem Cell and Regenerative Research, Tohoku University Graduate School of Dentistry, Sendai, Miyagi, Japan.*

Keywords: Cytokines, extracellular matrix, gingivitis, inflammation, mechanotransduction, mucosal immunity

*** Address corresponding to:**
Masahiro Yamada, DDS, PhD
Division of Molecular and Regenerative Prosthodontics,
Tohoku University Graduate School of Dentistry
4-1 Seiryo-machi, Aoba-ku, Sendai, 980-8575, Japan
tel: +81-22-717-8363; fax: +81-22-717-8367
E-mail: masahiro.yamada.a2@tohoku.ac.jp

Hiroshi Egusa, DDS, PhD
Division of Molecular and Regenerative Prosthodontics,
Tohoku University Graduate School of Dentistry
4-1 Seiryo-machi, Aoba-ku, Sendai, 980-8575, Japan
tel: +81-22-717-8363; fax: +81-22-717-8367
E-mail: egu@tohoku.ac.jp

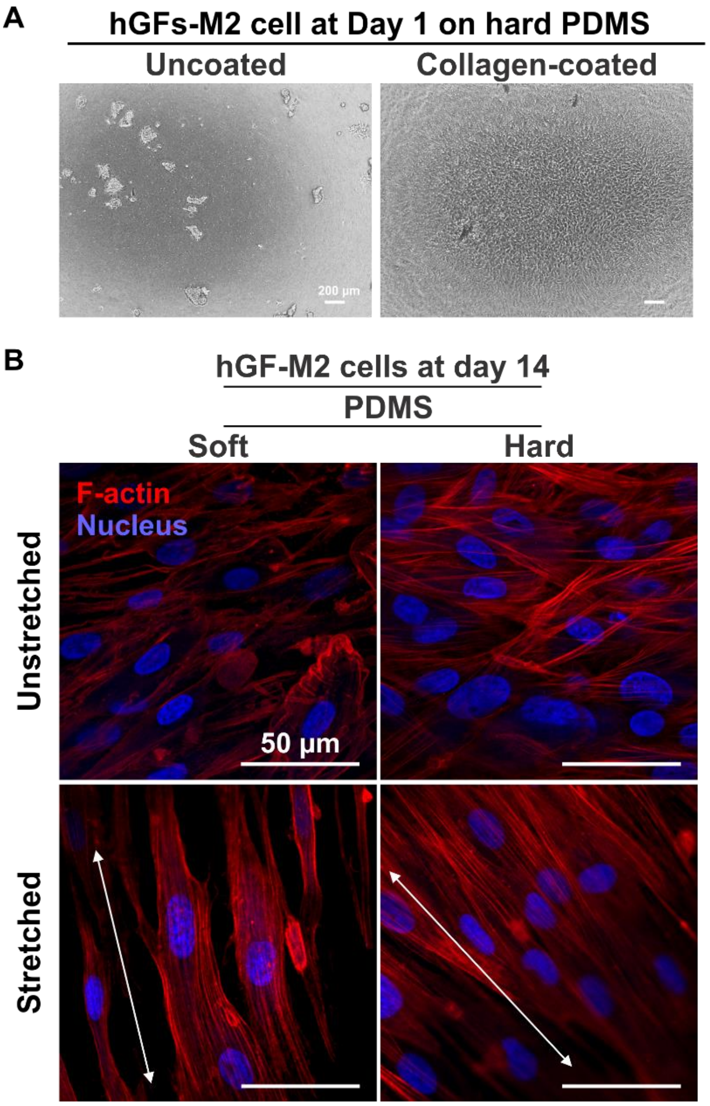

Supplementary Figure 1. Effects of collagen-coating on hGFs attachment on PDMS substrates

(**A**) Representative phase microscopic images in hGF-M2 cells cultured for 24 h on hard PDMS precoated with or without 0.1wt% collagen. (**B**) Fluorescence confocal laser microscopic images of *F*-actin (red), and nucleus (blue) in hGF-M2 cells cultured for 14 days on 0.1wt% collagen-coated soft or hard PDMS before and after unidirectionally stretching the PDMS substrates. Note: (**A**) cells cannot adhere to the PDMS substrate without the collagen coating (left panel) and (**B**) the hGFs are stretched along the direction axis of the PDMS substrates at day 14, regardless of the stiffness. Double-headed arrows in (**B**) indicate the stretching direction of PDMS substrates. hGFs, Human gingival fibroblasts; PDMS, Polydimethylsiloxane.

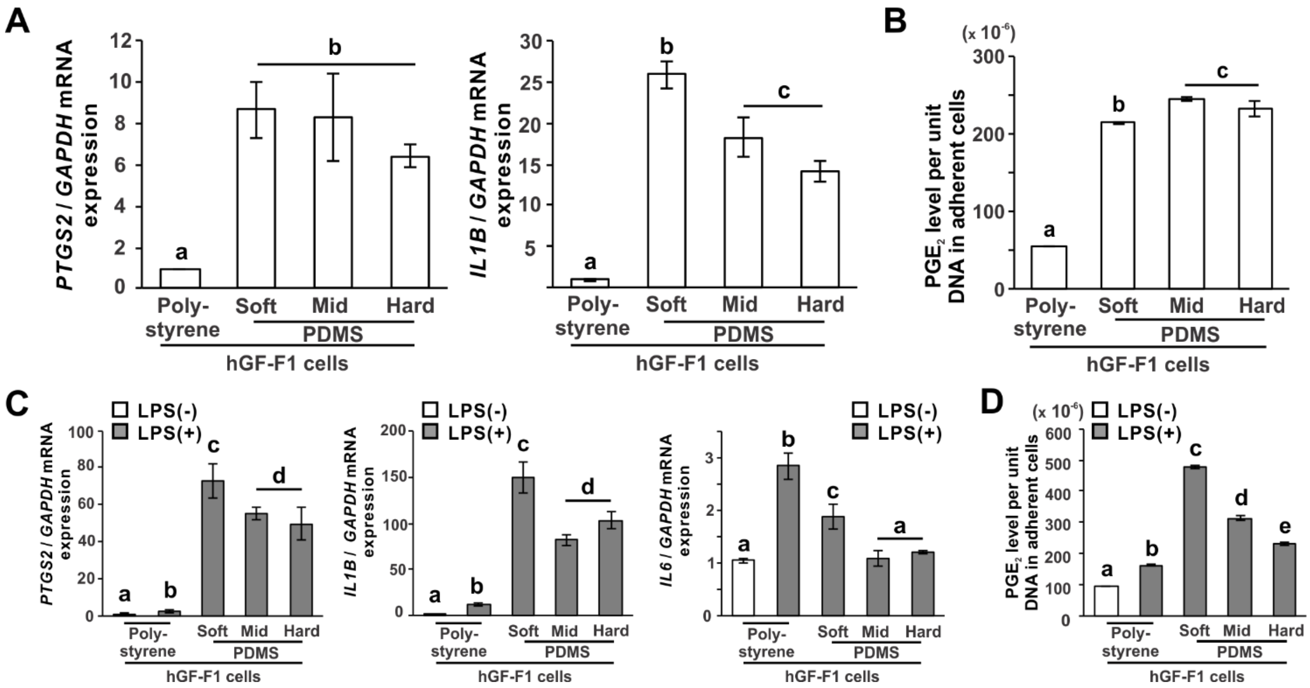

Supplementary Figure 2. Effects of substrate stiffness on proinflammatory responses in the other cell populations of hGFs under non-inflammatory or inflammatory condition

(**A** and **C**) Reverse transcription-polymerase chain reaction (RT-PCR)-based gene expression analysis of *PTGS2* and *IL1B* relative to *GAPDH* in hGF-F1 cells cultured on the 0.1wt% collagen-coated polystyrene culture plate and soft, mid, and hard PDMS for 12 h with or without co-incubation with 1,000 ng/mL of LPS. (**B** and **D**) PGE_2 levels per unit DNA in adherent hGF-F1 cells under the corresponding culture conditions described above by enzyme-linked immunosorbent assay (ELISA) analysis of culture supernatants. Data are represented as the mean ± standard deviation (SD; $N = 3$). Different letters indicate the statistically significant differences between them ($P < 0.05$; Tukey's honest significant difference [HSD] test). hGFs, Human gingival fibroblasts; LPS, Lipopolysaccharide; PDMS, Polydimethylsiloxane; PGE_2, Prostaglandin E2; *PTGS2*, Prostaglandin G/H synthase 2; *IL1B*, Interleukin-1β; *IL6*, Interleukin-6; *GAPDH*, Glyceraldehyde-3-phosphate dehydrogenase.

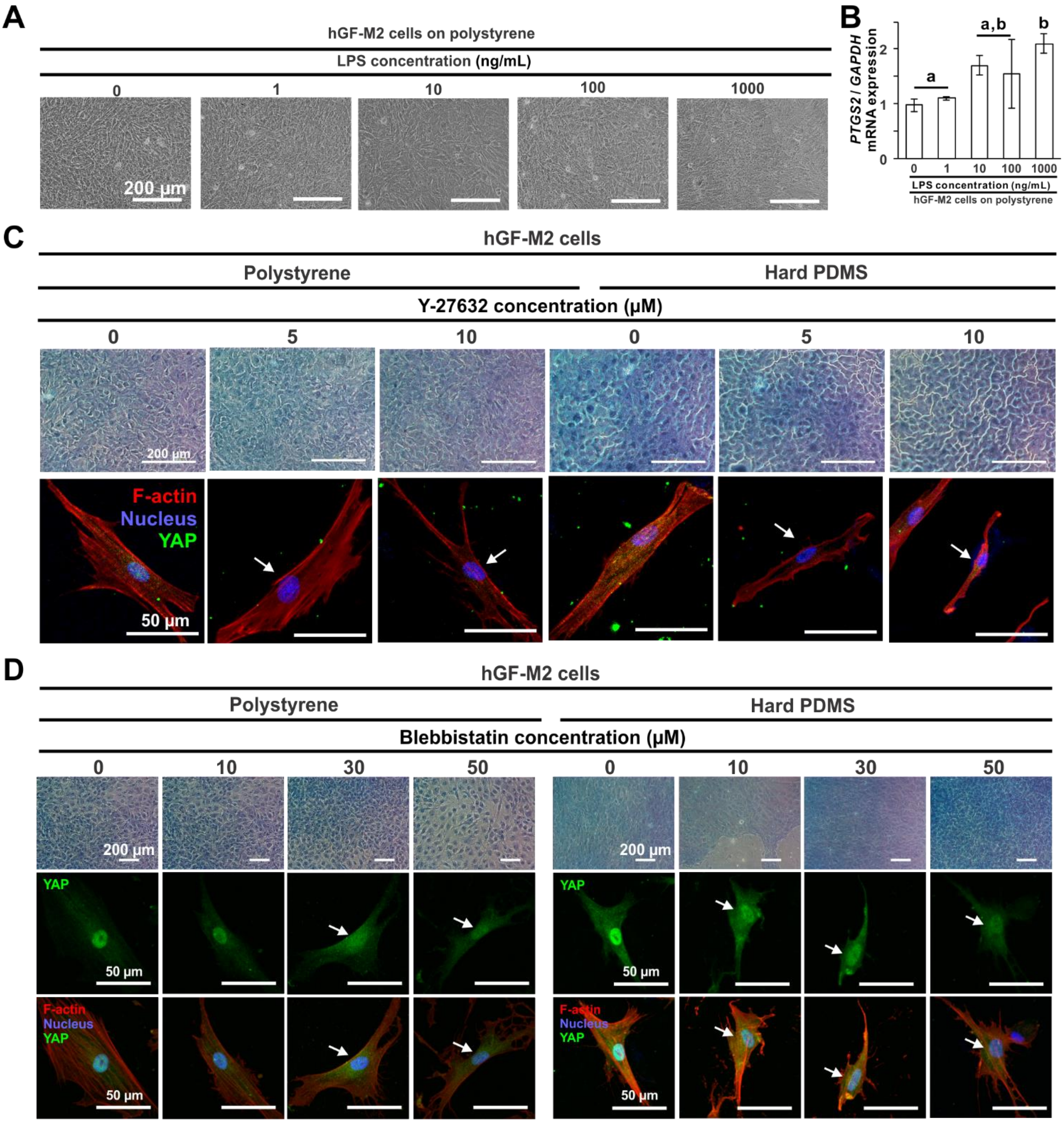

Supplementary Figure 3. Optimization for the concentration of LPS and mechanotransduction inhibitors in hGFs

(**A**) Representative phase microscopic images and (**B**) reverse transcription-polymerase chain reaction (RT-PCR)-based gene expression analysis of *PTGS2* relative to *GAPDH* in hGF-M2 cells cultured on a polystyrene culture plate after co-incubation with 0, 10, 100, and 1,000 ng/mL of LPS for 12 h. (**C** and **D**) Representative light microscopic images after methylene blue staining (upper images) and

immunofluorescence confocal laser microscopic images of YAP (green), *F*-actin (red), and/or nucleus (blue) (mid or bottom images) in hGF-M2 cells cultured on 0.1wt% collagen-coated polystyrene or hard PDMS for a total of 24 h, including 2 h of co-incubation with 0, 5, and 10 μM Y-27632 (**C**) or 0, 10, 30, and 50 μM blebbistatin (**D**). Note that 1000 ng/mL LPS induced the highest *PTGS2* expression in the hGF cell culture (**B**) without loss of cell attachment (**A**) and immunofluorescence confocal laser microscopic images show less or reduced YAP signals in the nucleus of hGF-M2 cells on both substrates after co-incubation with 5 μM or more Y-27632 (**C**, arrows) or 30 μM or more blebbistatin (**D**, arrows), respectively. Data are represented as the mean ± standard deviation (SD; $N = 3$). Different letters indicate the statistically significant differences between them ($P < 0.05$; Tukey's honest significant difference [HSD] test). hGFs, Human gingival fibroblasts; PDMS, Polydimethylsiloxane; LPS, Lipopolysaccharide; PTGS2, Prostaglandin G/H synthase 2; GAPDH, Glyceraldehyde-3-phosphate dehydrogenase; ROCK, Rho-associated coiled-coil containing protein kinase; YAP, Yes-associated protein.

Encoded protein name (gene name)	Primers (Fw, forward; Rv, reverse)	Product size (bp)	Accession number
**Human**			
Prostaglandin G/H synthase 2 *(PTGS2)*	Fw: 5'-TCCAGTACCAAAATCGTATTGCT-3'<br>Rv: 5'-AGTGCTTCCAACTCTGCAGACAT-3'	370	NM_000963.4
Interleukin-1β *(IL1B)*	Fw: 5'-TGGAGCAACAAGTGGTGT-3'<br>Rv: 5'-TTGGGATCTACACTCTCCAGC-3'	157	NM_000576.3
Interleukin-6 *(IL6)*	Fw: 5'-TCAATGAGGAGACTTGCCTG-3'<br>Rv: 5'-GATGAGTTGTCATGTCCTGC-3'	261	NM_001371096.1
Nitric oxide synthase 2 *(NOS2)*	Fw: 5'-CAGCGGGATGACTTTCCAA-3'<br>Rv: 5'-AGGCAAGATTTGGACCTGCA-3'	75	NM_000625.4
Tumor Necrosis Factor-α *(TNFA)*	Fw: 5'-GAGGCCAAGCCCTGGTATG-3'<br>Rv: 5'-CGGGCCGATTGATCTCAGC-3'	91	NM_000594.4
Collagen Type 1 Alpha 1 *(COL1A1)*	Fw: 5'-AGGCGAACAGGGCGACAGAG-3'<br>Rv: 5'-GGCCAGGGAGACCGTTGAGT-3'	185	NM_000088.4
Elastin *(ELN)*	Fw: 5'- AACCAGCCTTGCCCGC-3'<br>Rv: 5'- CCCCAAGCTGCCTGGTG-3'	101	NM_001278939.2
Fibrillin 1 *(FBN1)*	Fw: 5'-GAATGCAAGAACCTCATTGGCAC-3'<br>Rv: 5'-TGGCGGTAAACCCATCATTACAC-3'	184	NM_000138.5
Lysyl Oxidase *(LOX)*	Fw: 5'-AGCATACAGGGCAGATGTCAGAG-3'<br>Rv: 5'-CTTGGTCGGCTGGGTAAGAAAT-3'	105	NM_002317.7
Paxillin *(PXN)*	Fw: 5'-CCCTGACGAAAGAGAAGCCTAAG-3'<br>Rv: 5'-AGATGCGTGTCTGCTGTTGG-3'	186	NM_001385988.1
Integrin β-1 *(ITGB1)*	Fw: 5'-TTTGTTTAATGTCTGGTGCTTTCTG-3'<br>Rv: 5'-CCCCAAAATTGCAAACAAATACA-3'	69	NM_133376.3
Integrin α-11 *(ITGA11)*	Fw: 5'-CAGCTCGCTGGAGAGATACG-3'<br>Rv: 5'-TTACAGGACGTGTTCGCCTC-3'	186	NM_001004439.2
Glyceraldehyde 3 Phosphate Dehydrogenase *(GAPDH)*	Fw: 5'-AATCCCATCACCATCTTCCA-3'<br>Rv: 5'-TGGACTCCACGACGTACTCA-3'	82	NM_001357943.2

Supplementary Table 1. List of primers for human genes used in SYBR-green-based polymerase chain reaction.
