# Supplementary material for: Substrate stiffness controls proinflammatory responses in human gingival fibroblasts
Source: Sci Rep. 2023 Jan 24;13:1358. doi: 10.1038/s41598-023-28541-z (PMC9873657; doi:10.1038/s41598-023-28541-z)

Fig 4B: p-YAP 397

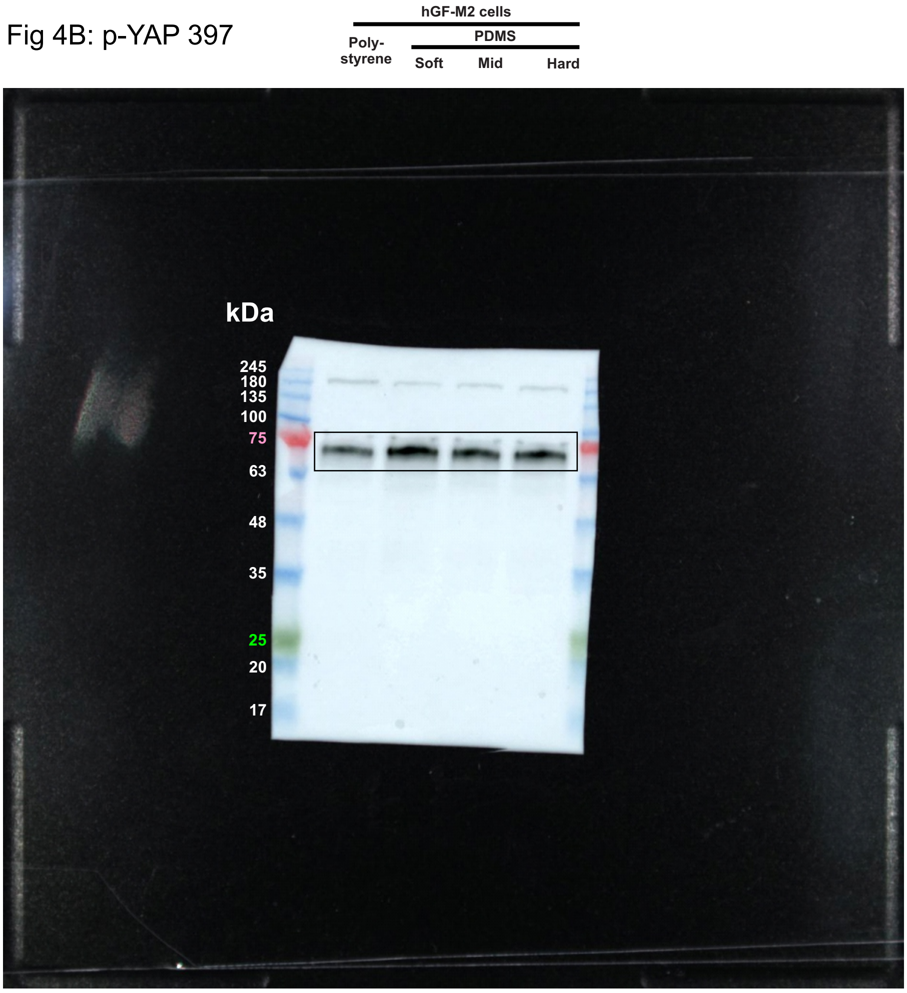

Fig. 4B: p-YAP 127

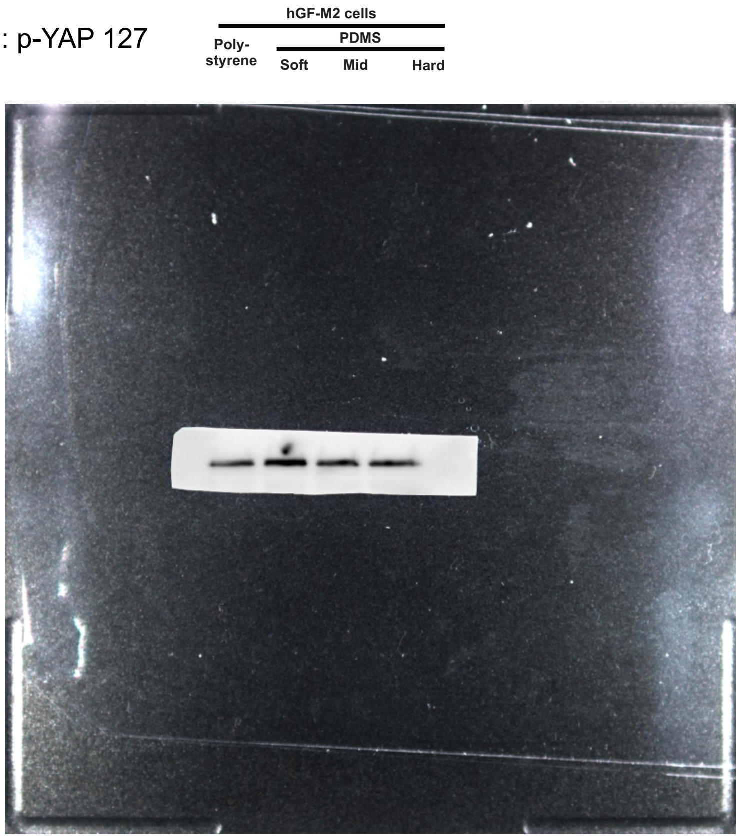

Fig. 4B: active-YAP

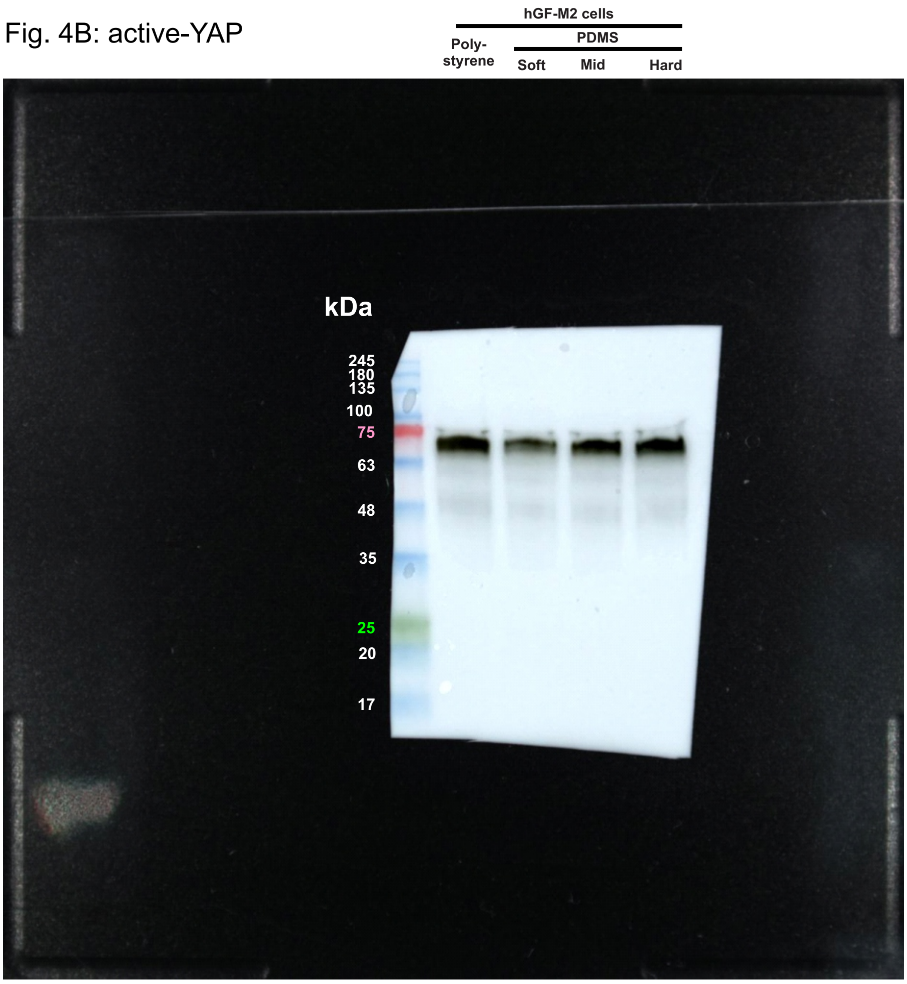

Fig 4B: total-YAP

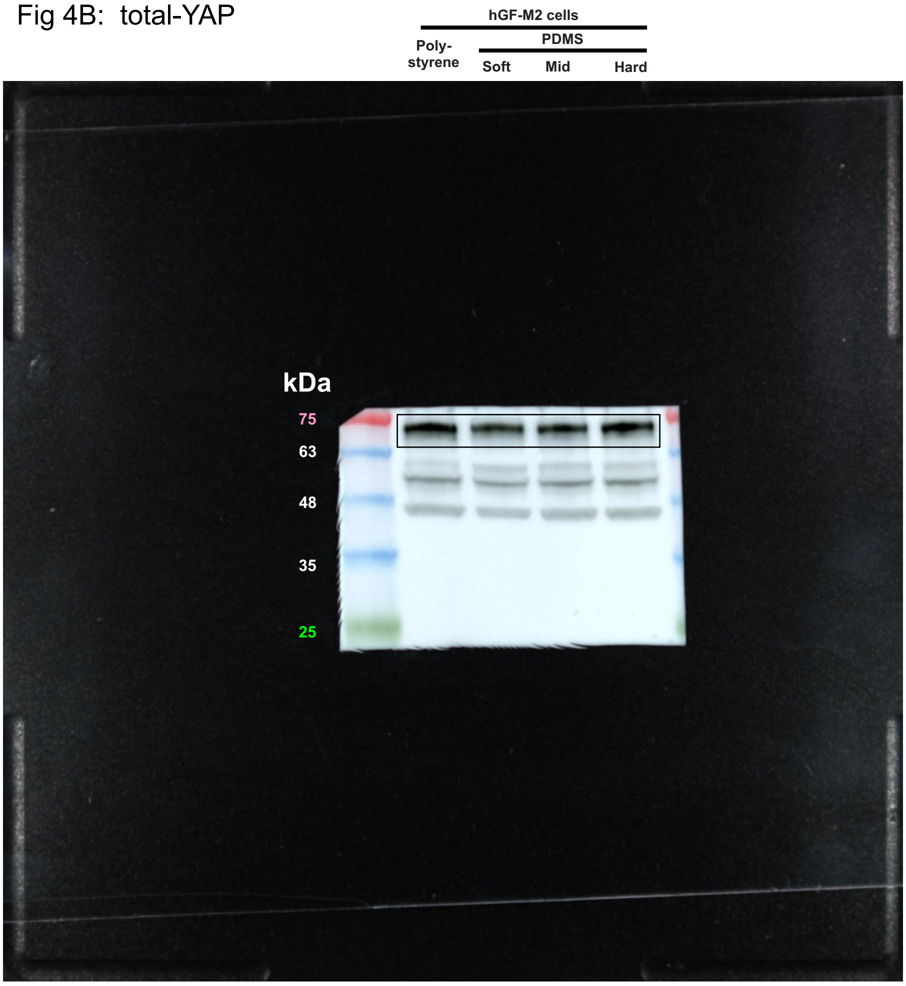

Fig. 4B: GAPDH

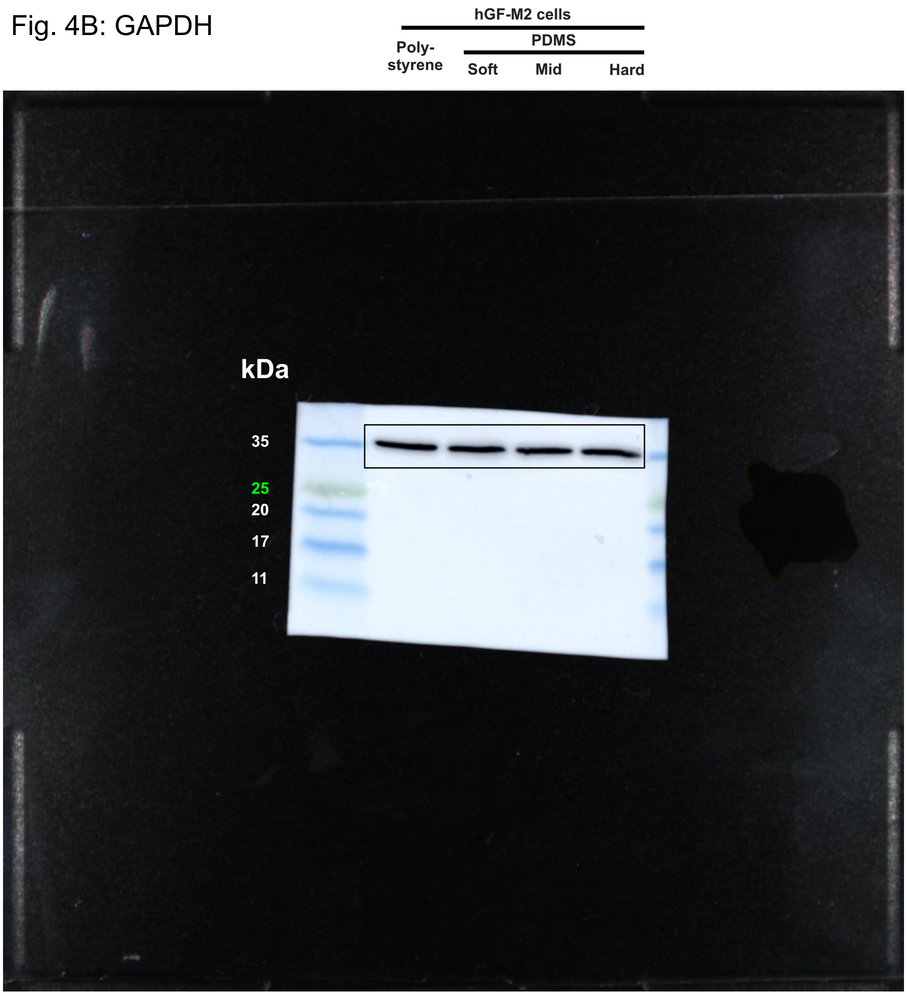

Fig. 4C: p-NF-kB

hGF-M2 cells

Poly-styrene | PDMS: Soft, Mid, Hard

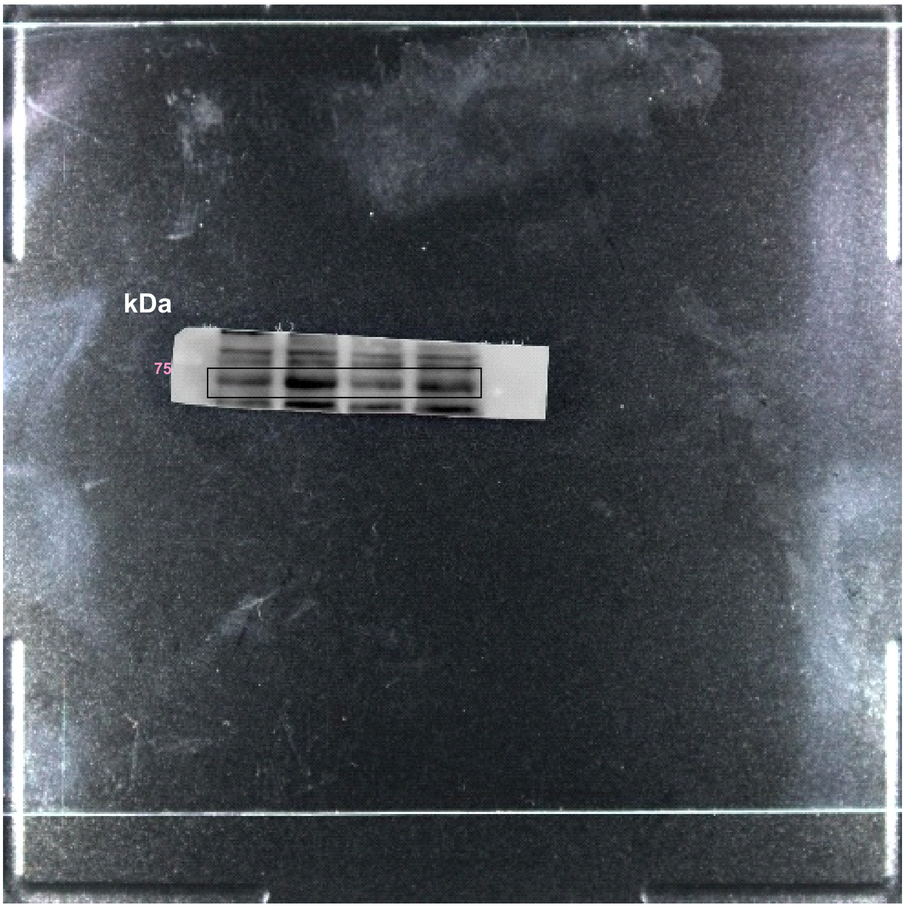

Fig. 4C: Total-NF-kB

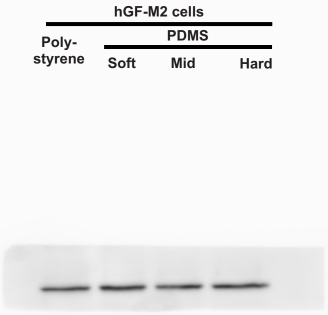

Fig. 4C: GAPDH

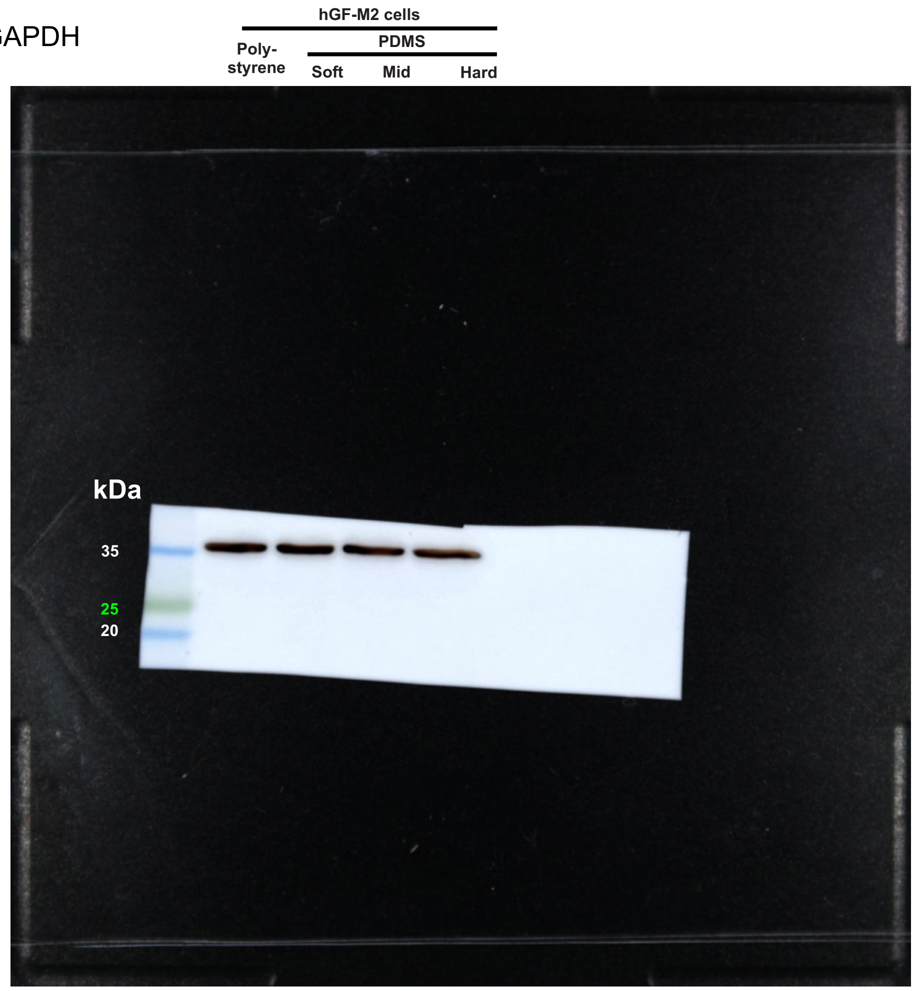

Fig. 4D: p-NF-kB (+LPS)

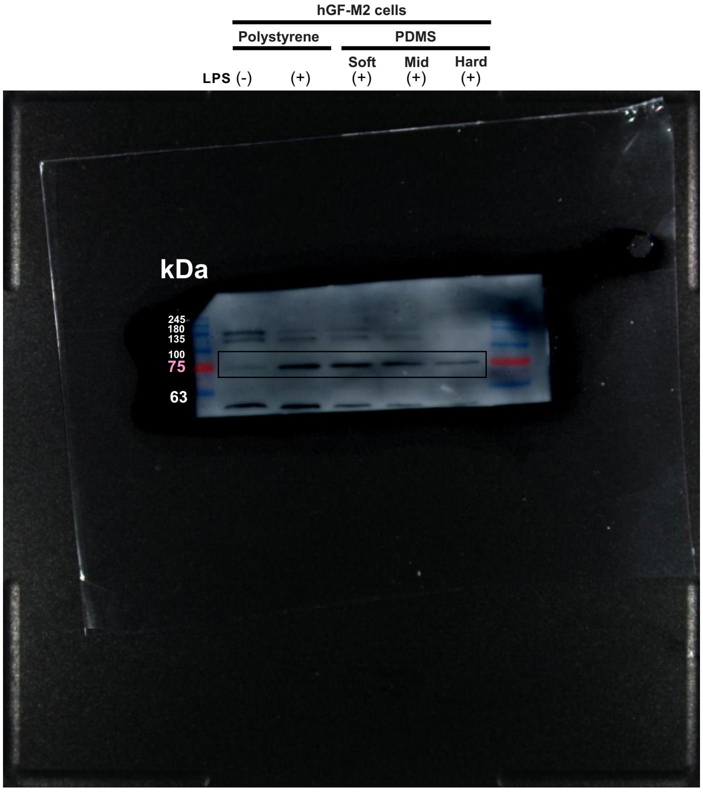

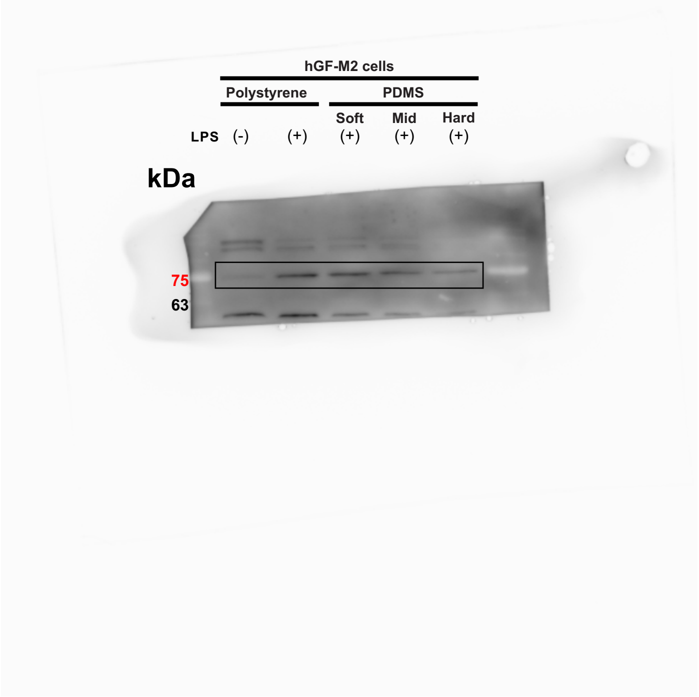

Fig. 4D: Total-NF-kB +LPS

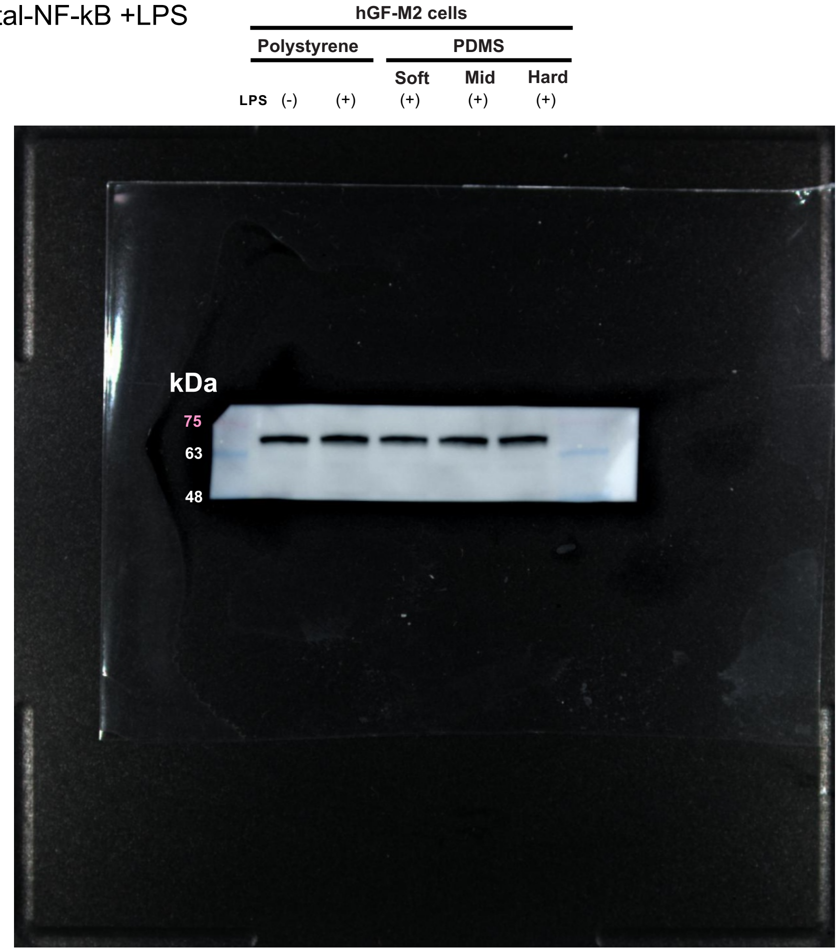

Fig. 6G: TLR-4

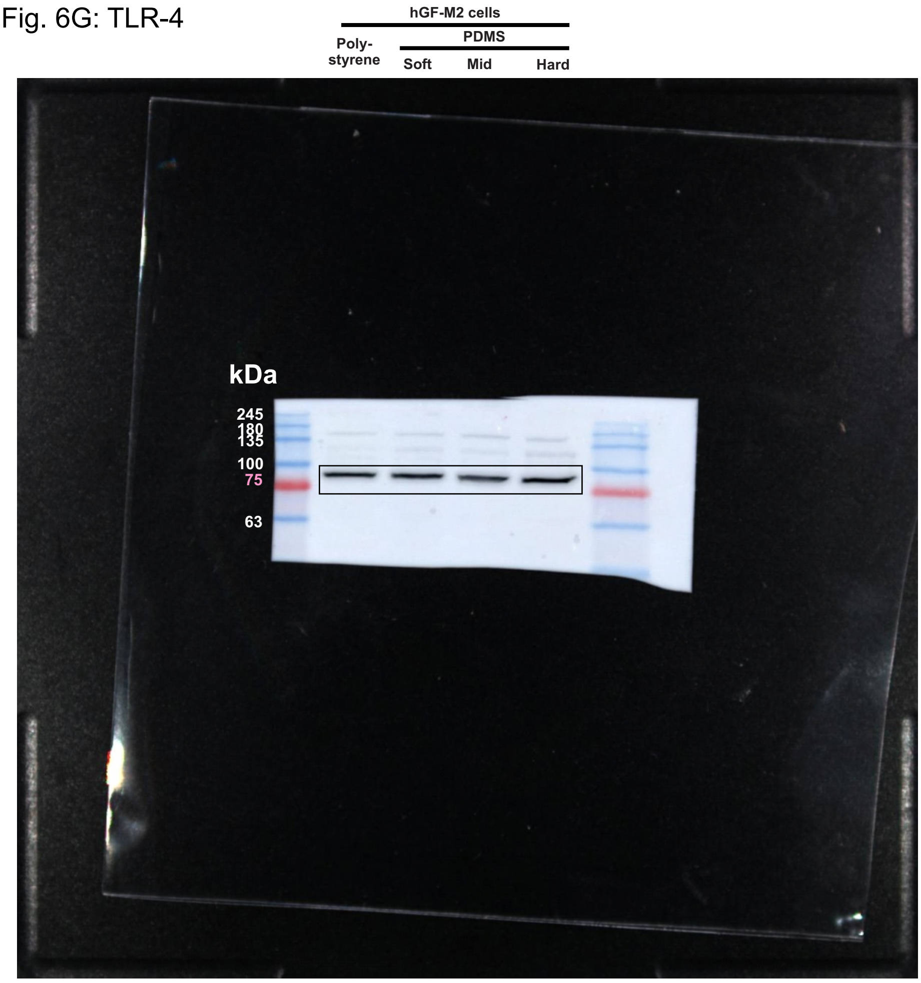

hGF-M2 cells
Poly-
styrene
PDMS
Soft
Mid
Hard
kDa
245
180
135
100
75
63

Fig. 4D&6H: GAPDH +LPS

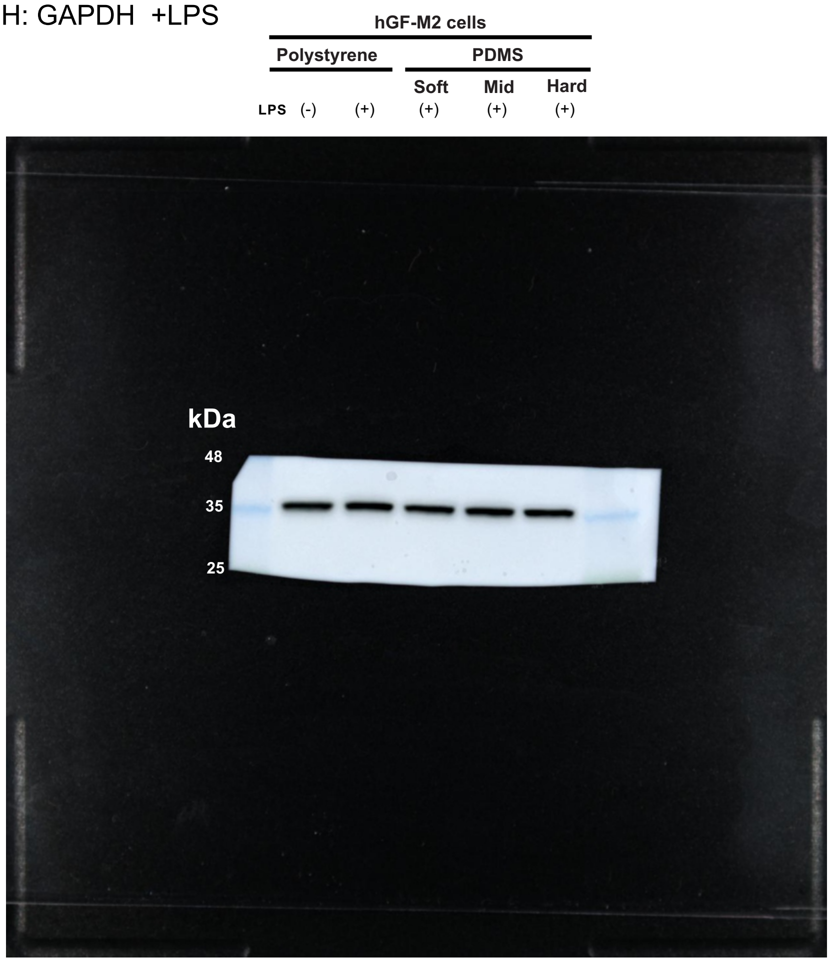

Fig. 6G: GAPDH

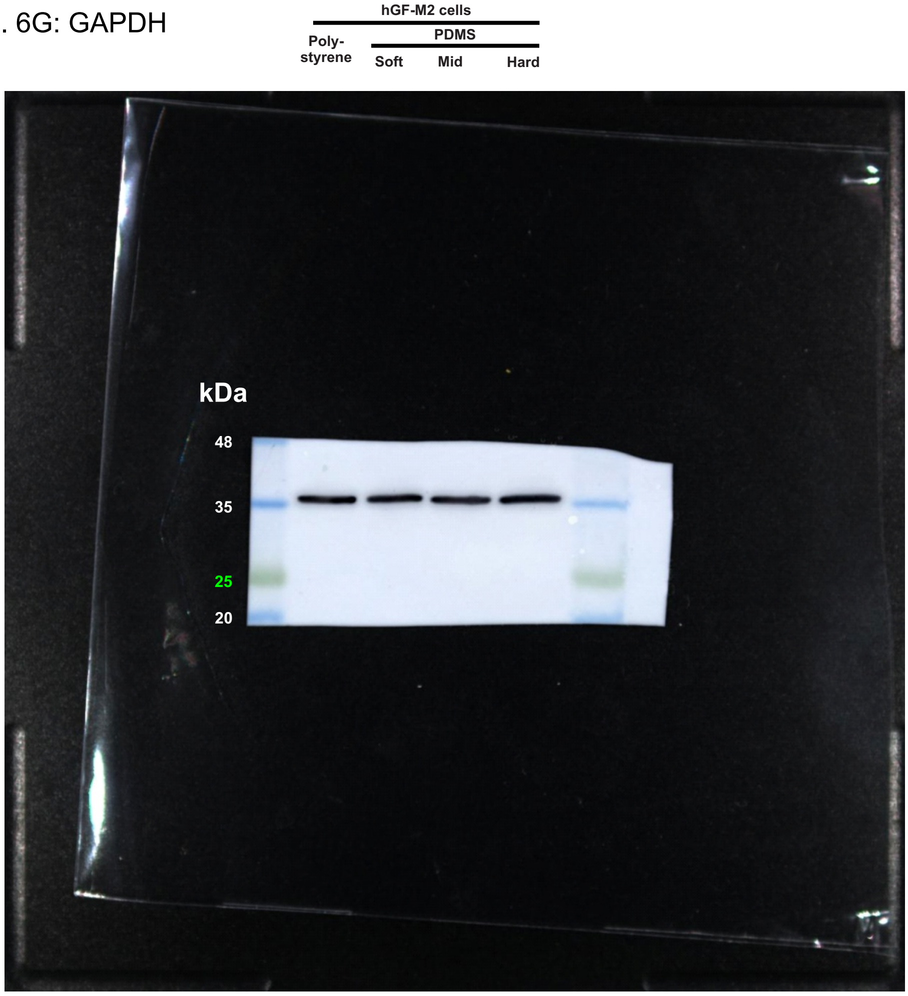

Fig. 6H: TLR-4 (+LPS)

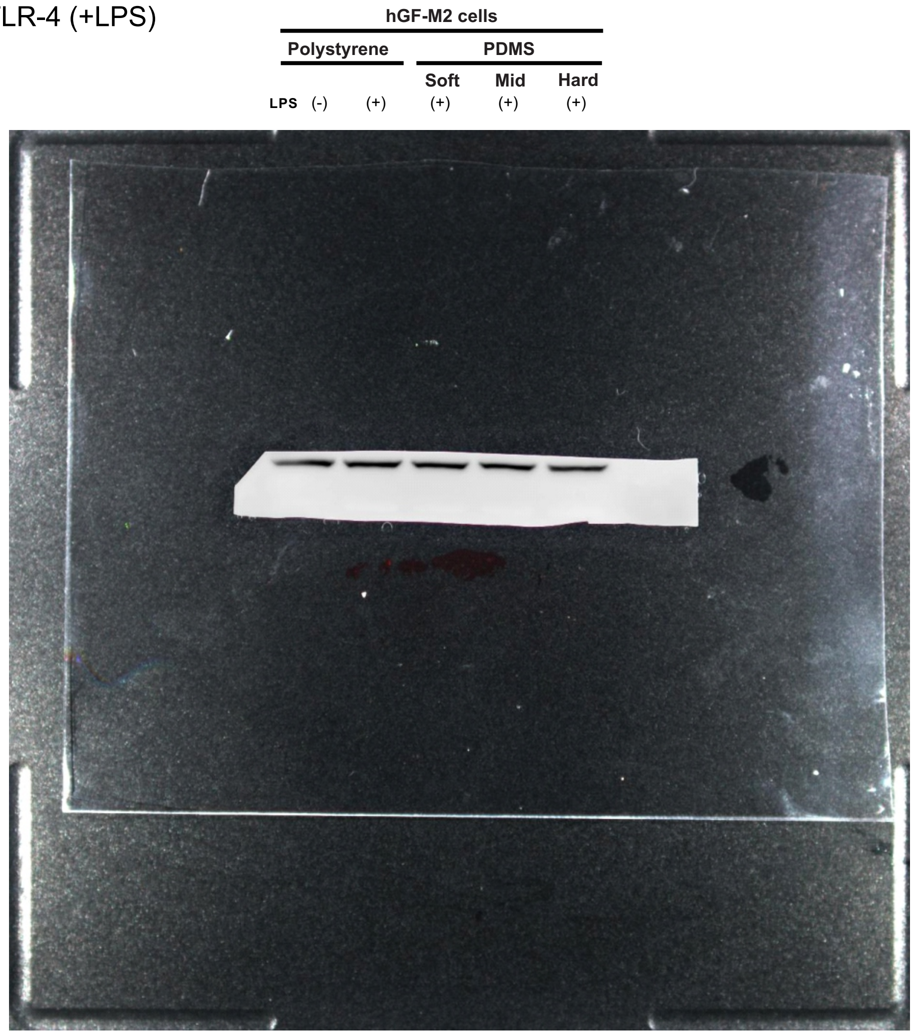

Supplement: Supplementary file 2 — Supplementary Information 2. [file 41598_2023_28541_MOESM2_ESM.pdf]
